# Gas-stabilizing nanoparticles for ultrasound imaging and therapy of cancer

**DOI:** 10.1186/s40580-021-00287-2

**Published:** 2021-12-01

**Authors:** Sinan Sabuncu, Adem Yildirim

**Affiliations:** CEDAR, Knight Cancer Institute, School of Medicine, Oregon Health & Science University, Portland, OR 97201 USA

**Keywords:** Ultrasound imaging, Focused ultrasound, Gas-stabilizing nanoparticles, Tumor ablation, Drug delivery, Sonodynamic therapy, Cancer theranostics

## Abstract

The use of ultrasound in the clinic has been long established for cancer detection and image-guided tissue biopsies. In addition, ultrasound-based methods have been widely explored to develop more effective cancer therapies such as localized drug delivery, sonodynamic therapy, and focused ultrasound surgery. Stabilized fluorocarbon microbubbles have been in use as contrast agents for ultrasound imaging in the clinic for several decades. It is also known that microbubble cavitation could generate thermal, mechanical, and chemical effects in the tissue to improve ultrasound-based therapies. However, the large size, poor stability, and short-term cavitation activity of microbubbles limit their applications in cancer imaging and therapy. This review will focus on an alternative type of ultrasound responsive material; gas-stabilizing nanoparticles, which can address the limitations of microbubbles with their nanoscale size, robustness, and high cavitation activity. This review will be of interest to researchers who wish to explore new agents to develop improved methods for molecular ultrasound imaging and therapy of cancer.

## Introduction

Ultrasound imaging has been used in the clinic for several decades for cancer detection and diagnosis and image guidance during tissue biopsies [1–3]. It poses several advantages such as low-cost, real-time imaging capability, non-ionizing nature, and deep tissue penetration over other common medical imaging modalities. Ultrasound based methods have also been developed for cancer therapy. For example, high intensity focused ultrasound (HIFU) can be used to ablate solid tumors by generating a rapid temperature increase in the treated tissue [4, 5]. This emerging technology has been recently approved by The United States Food and Drug Administration (FDA) for the treatment of uterine fibroids and prostate tissue [6, 7]. In addition, several ongoing clinical trials worldwide are testing this method in the ablation of other solid tumors, including breast, pancreas, and brain cancers. Other ultrasound applications in cancer therapy include sonodynamic therapy, localized drug delivery, and blood–brain-barrier opening [2, 8–13].

Ultrasound contrast agents were first developed more than 50 years ago for contrast enhanced ultrasound imaging using stabilized air ‘microbubbles’ [14–16]. Today, microbubbles (~ 1–10 µm) are typically produced by coating perfluorocarbon bubbles with a stabilizing shell such as phospholipid, polymer, or protein layers [17]. Several clinically approved microbubble compositions are commercially available worldwide for mainly cardiac applications [15, 18]. Microbubbles can improve the contrast of ultrasound images by effectively backscattering ultrasound pulses and generating harmonic frequencies resulting from stable bubble cavitation (i.e., periodic contraction and expansion of a bubble in response to ultrasound waves with positive and negative pressures) [14]. When ultrasound pulses at higher acoustic intensities are applied, bubble oscillation becomes unstable, yielding bubble collapse (i.e., inertial cavitation). As a result, mechanical effects such as shock waves, water jets, and shear forces are produced, which can improve the outcomes of ultrasound therapies by ablating cells, porating cell membranes, and inducing cell apoptosis or necrosis [13, 19]. In addition, microbubble surfaces can be modified with targeting molecules to develop molecular imaging agents or targeted therapies. However, the large size of microbubbles limits their applications to the vascular space as they cannot extravasate from blood vessels [20]. In addition, microbubbles demonstrate poor stability in circulation, with half-lives on the order of minutes. Finally, microbubbles can quickly be destroyed by high-intensity ultrasound pulses typically utilized in ultrasound therapies; thus, they cannot provide durable inertial cavitation activity for long periods of time [20, 21].

In the light of these limitations of microbubbles, there is ongoing research to develop smaller ultrasound contrast agents, preferably < 200 nm, and with better stability in circulation. Such nanoscale and robust contrast agents may accumulate in solid tumors through enhanced permeability and retention (EPR) effect and transcytosis (i.e., nanoparticle transport into tumors by endothelial cells) [22, 23]. It should be noted that the nanoparticle uptake may vastly vary between tumor types and the exact mechanism of tumor accumulation is still under investigation. Nevertheless, a large body of research evidence suggests that nanoparticles with suitable physical and chemical properties (e.g., surface charge, particle size, and shape) can effectively accumulate in solid tumors [24–26].

The initial result of the search for nanoscale contrast agents was the development of phase change fluorocarbon droplets with submicrometer sizes [27]. When irradiated using high intensity ultrasound pulses, the volatile cores of nanodroplets can vaporize to form micron-sized bubbles (i.e., acoustic droplet vaporization). Later, other sub-micrometer ultrasound contrast agents based on fluorocarbon gas-filled polymer or inorganic shells or using gas-generating nanoparticles [28–31]. However, most of these agents were still larger than 200 nm and/or demonstrated poor in vivo stability due to their fluorocarbon components. More information on such nanoscale ultrasound contrast agents could be found in previous comprehensive reviews [13, 20, 32, 33].

In this review, we will describe a distinct class of ultrasound responsive materials; gas stabilizing nanoparticles (GSNs) [34–38]. These solid nanoparticles are engineered to stabilize gas-pockets on their surfaces or inside their hydrophobic cavities, which can act as heterogeneous nucleation sites to produce micron-sized cavitating bubbles under ultrasound insonation [13, 38]. Recent studies showed that nanoscale GSNs with sizes down to 50 nm could be easily prepared as they do not have a fluorocarbon component [39]. Their fluorocarbon-free nature also provides them with exceptional signal stability during ambient storage for months and in vivo for several days after intravenous injection [34, 39, 40]. In addition, GSNs have been found to generate a stronger and more durable cavitation activity than nanodroplets or microbubbles, making them highly attractive for cancer theranostics applications [37, 41–43]. Below we will first discuss the design principles of GSNs. Then, we will detail the applications of GSNs in ultrasound imaging, tumor ablation, drug delivery, and sonodynamic therapy. Finally, we will conclude by discussing the current status and future potential of GSNs in cancer theranostics.

## Gas stabilizing nanoparticles (GSNs) as exogenous nuclei for acoustic cavitation

**Fig. 1:**
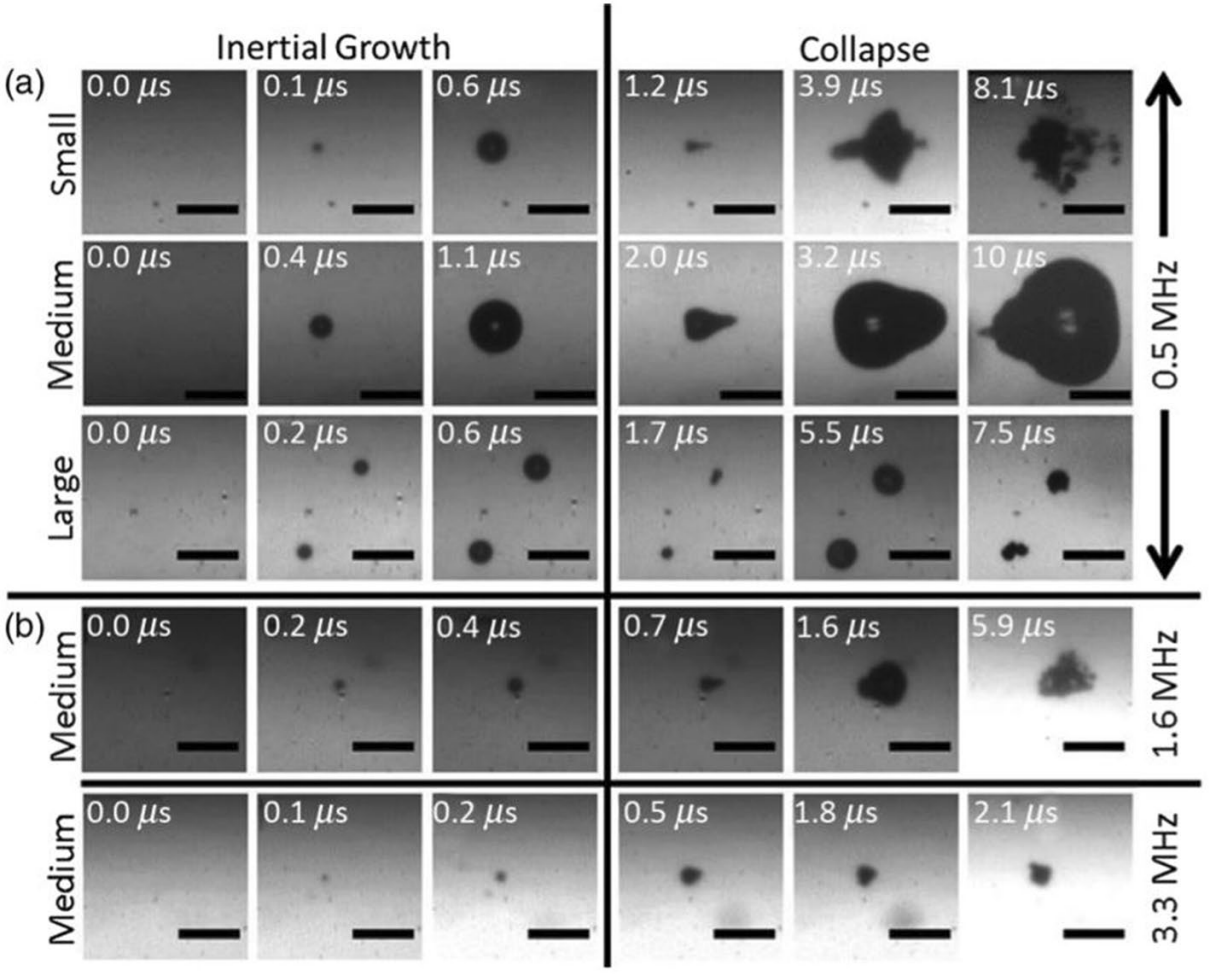
High speed camera observations of microbubbles generated by polymer nanocups with different sizes and under ultrasound insonation with a frequency of 0.5, 1.6, or 3.3 MHz. Adapted from reference [45], Copyright 2016, American Physical Society

In recent years several different types of GSNs have been designed and developed for theranostic ultrasound applications. These studies found that in the presence of GSNs, acoustic cavitation in water could be initiated using low-intensity ultrasound pulses, which generally do not cause cavitation in water, indicating that GSNs can act as exogenous nuclei for acoustic cavitation. During the acoustic cavitation process, micron-sized short-lived bubbles are generated. The growth and collapse of these bubbles under ultrasound insonation have been imaged using high-speed cameras (Fig. 1) [40, 44, 45]. These studies showed that bubble growth and collapse happen in the order of tens of microseconds. Depending on the acoustic parameters, these bubbles can reach sizes up to 100 µm before they collapse. For example, Jin et al. [44] found that the average bubble size was increased about 40% when the peak negative pressure (PNP) of HIFU pulses (2 MHz) was increased to 6 from 4 MPa. On the other hand, Kwan et al. [45] investigated the effect of ultrasound frequency on bubble size. They observed that the bubble diameter could exceed 100 µm at 0.5 MHz; however, it was only ~ 20 µm at 3.3 MHz. While these studies provide valuable insight into the effect of ultrasound parameters on cavitation bubble formation, it should be kept in mind that in biological environments, the bubble formation dynamics can be expected to be very different from these studies where cavitation was studied in homogenous solvents with no boundaries.

The micron-sized bubbles generated by GSNs can backscatter the ultrasound waves and improve the contrast of ultrasound images [34, 35, 39]. In addition, the violent collapse of these bubbles can induce mechanical effects (e.g., shock waves and water jets) in the tissue, which are useful for improving the outcomes of ultrasound-based therapies such as tumor ablation or localized drug delivery [37, 40, 46, 47]. Finally, the high temperatures and pressures reached inside the cavitation bubbles during collapse can result in reactive oxygen species (ROS) generation and, thus, enhanced sonodynamic effect [48, 49]. As a result of these features, GSNs have started to attract the attention of researchers for several applications in ultrasound imaging and therapy of cancer. Before moving to their specific applications in ultrasound theranostics, we will briefly review the potential mechanisms proposed for cavitation inception by GSNs and their most common types.

### Cavitation inception by solid nanoparticles

The earliest studies on cavitation of water date back to the eighteenth century [50, 51]. One of the critical findings of these studies was that cavitation inception always happens at a lower pressure than the theoretically calculated cavitation pressure of homogenous water and is highly dependent on the purity of the water used in the study [52–55]. These observations indicated the almost inevitable presence of heterogeneous nuclei in water, such as bubbles, surfactants, dirt particles, or clusters of organic molecules, which can lower the cavitation threshold [51, 53, 55].

One widely accepted theory to explain the heterogeneous nucleation of bubbles is the crevice model, where gas pockets stabilized inside the ‘crevices’ on dirt particles or container surfaces can act as heterogeneous nuclei for water cavitation [56, 57]. Likewise, solid particles with suitable surface properties and morphologies can potentially stabilize gas-pockets on their surfaces or cavities. In fact, several studies found that the presence of solid particles could reduce the cavitation threshold of water [58–61]. Most studies in this field hypothesized that gas-pockets (i.e., nanobubbles) stabilized on particle surfaces or their pores/cavities act as heterogeneous nuclei for acoustic cavitation. However, the question of how solid particles incept cavitation has not been fully answered yet.

**Fig. 2:**
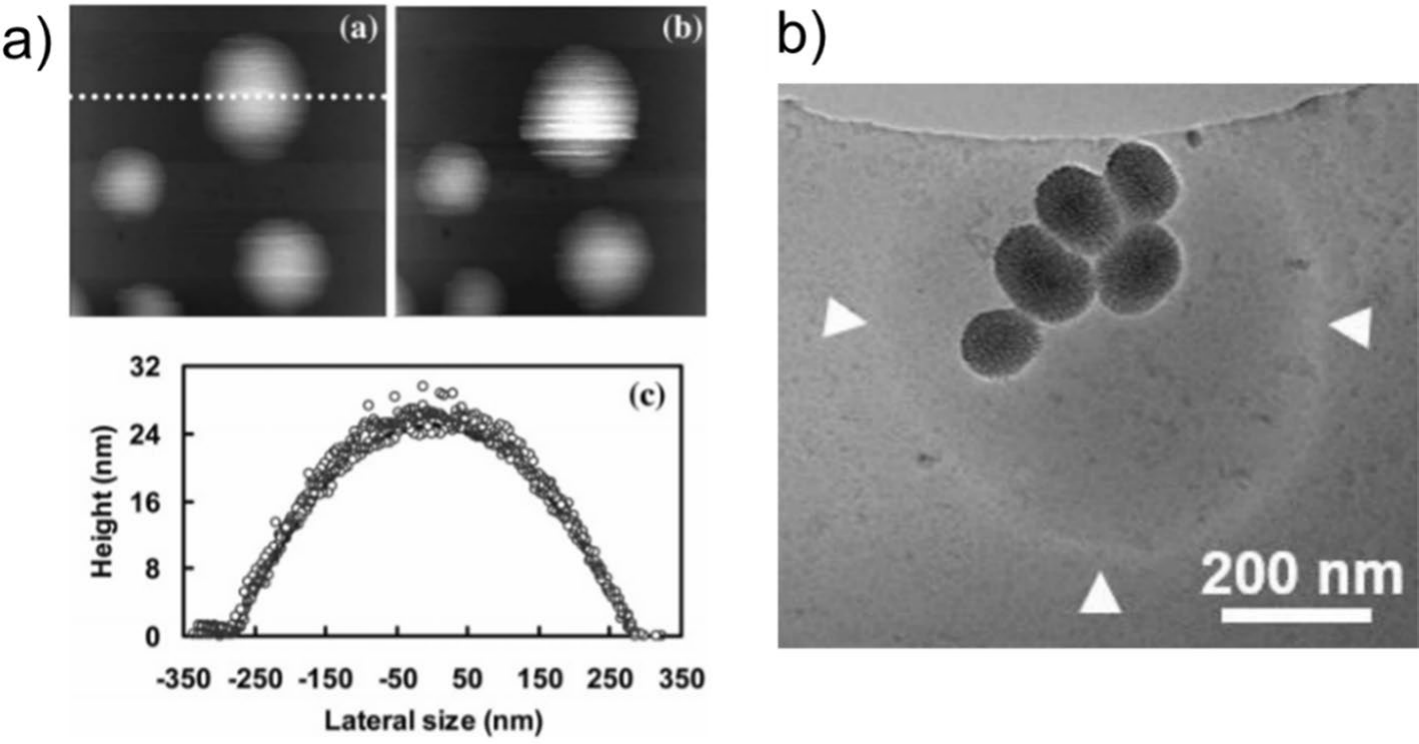
**a** AFM images of nanobubbles formed on an octadecyltrichlorosilane modified silicon substrate. Adapted from reference [65], Copyright 2006, American Chemical Society, **b** Cryo-EM image of a bubble stabilized by hydrophobic MSNs. Adapted from reference [73], Copyright 2021, John Wiley and Sons

Free-standing nanobubbles are unstable in water due to the extremely high interfacial pressures at the water–gas interface, and thus, they quickly dissolve in less than a second [62]. Nanobubbles attached to a surface, on the other hand, were found to be stable for several weeks at ambient conditions [63, 64] and were imaged using different microscopy techniques such as atomic force microscopy (AFM) or total internal reflection fluorescence (TIRF) microscopy (Fig. 2a) [65, 66]. In addition, cavitation inception by surface stabilized nanobubbles has been demonstrated experimentally [67–69]. Thus, these observations with surface nanobubbles suggest that nanoparticles with suitable surface properties could stabilize nanobubbles with cavitation inception ability.

Another possible mechanism for acoustic cavitation initiation by nanoparticles is the formation of gas-rich layers around nanoparticles as a result of gas enrichment on the hydrophobic interface [70]. Similar to the nanobubble hypothesis, this theory is also based on the former studies performed on surfaces, where the formation of gas-rich layers, also called nano/micro pancakes, was imaged on hydrophobic surfaces. As the name implies, gas-rich layers have higher aspect ratios than nanobubbles with typically larger diameters and smaller heights [71, 72]. Yeh and coworkers [70] proposed that gas-rich layers may be present on nanoparticle surfaces. As stated by the authors, it is also possible that gas-rich layers can coexist with nanobubbles on the nanoparticle surface, both of which can reduce the acoustic pressures required for bubble nucleation and growth.

Finally, a recent study by Liu and coworkers proposed that nanoparticles can adsorb to the surface of bubbles present in water and stabilize them by reducing the interfacial energy [73]. Different from the above, in this case, multiple nanoparticles stabilize one larger bubble. The authors imaged submicrometer bubbles stabilized by hydrophobically modified mesoporous silica nanoparticles (MSNs) using cryogenic electron microscopy (Cryo-EM) (Fig. 2b). To further prove the presence of stabilized bubbles in nanoparticle colloids, they performed nanoparticle tracking analysis (NTA) studies, where they added a small amount of ethanol to destabilize the bubbles. They observed that after ethanol addition, average particle size was reduced as a result of bubble dissolution. Overall, further studies are needed to better understand which one(s) of these proposed mechanisms contribute to the acoustic cavitation inception by engineered nanoparticles.

### Design of GSNs for ultrasound theranostics

In a series of studies from 2001 to 2005, Esenaliev and coworkers demonstrated improved drug delivery to solid tumors in the presence of polystyrene particles (100–300 nm) and ultrasound insonation with a frequency of 20 kHz [74–76]. However, in these studies, commercially available polystyrene spheres have been directly used without any specific modification. To our knowledge, the first report on rationally designing a particle to stabilize nanobubbles for improved cavitation activity is the study by Coussios and coworkers [77]. In this study, the authors decorated polystyrene microparticles (300 or 600 nm) with silica nanoparticles (20 nm) to generate a rough surface, making the nanobubble stabilization on the surface more favorable. The authors reported a cavitation threshold of 5 MPa (PNP) for water under HIFU insonation at a frequency of 1.1 MHz. In the presence of smooth or rough surface 600 nm polystyrene particles, the cavitation threshold reduced to 2.5 MPa or 0.5 MPa, respectively, suggesting that roughness can improve the cavitation activity of solid particles.

**Fig. 3:**
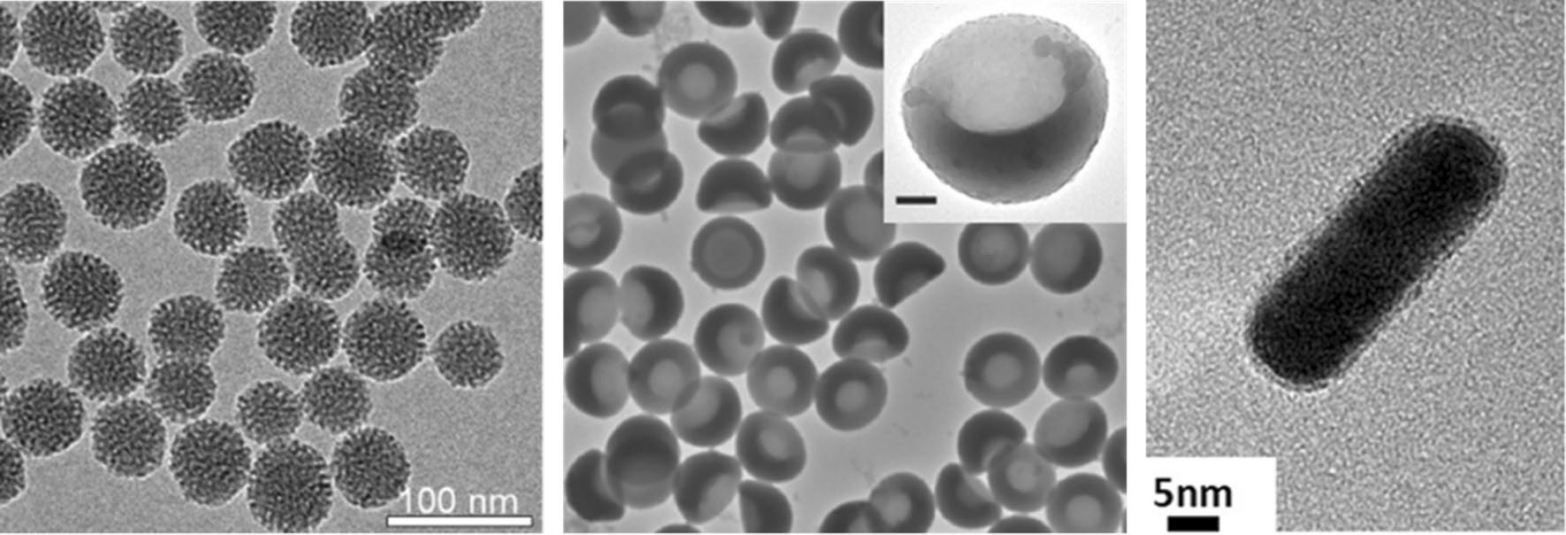
TEM images of different GSNs. Pluronic F127 polymer coated hydrophobic MSNs (left), polymer nanocups (middle, scale bar is 100 nm), and peptide hydrogel coated gold nanorods (right). Adapted from references [37, 39, 84]. https://pubs.acs.org/doi/10.1021/acsomega.0c03377, 2020, further permissions related to the material excerpted should be directed to the American Chemical Society. Copyright 2015, John Wiley and Sons, Copyright 2019, American Chemical Society, respectively

**Fig. 4:**
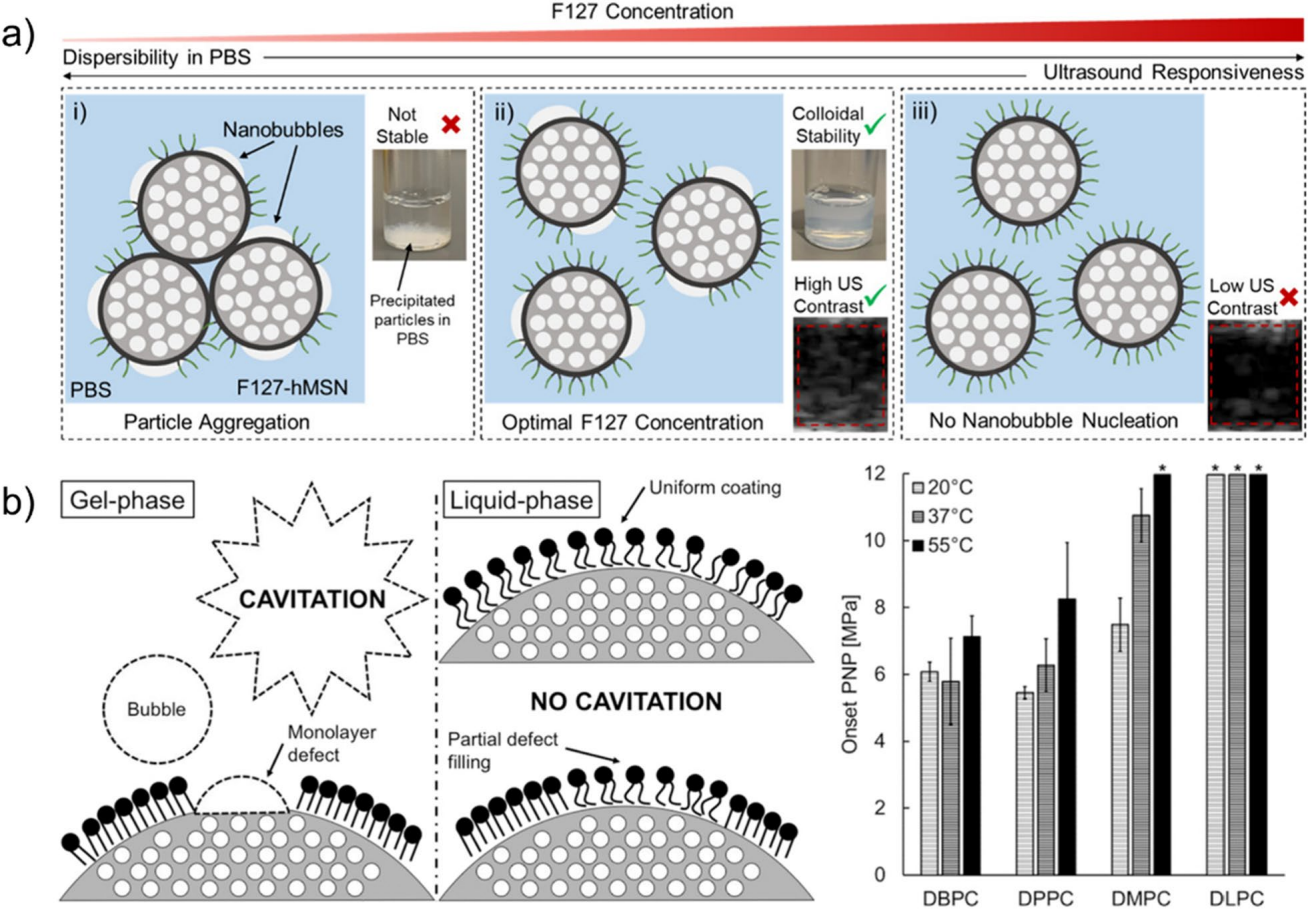
**a** Schematic of the proposed mechanism for GSNs with high acoustic activity and good dispersibility in buffer solutions. Adapted from reference [39], https://pubs.acs.org/doi/10.1021/acsomega.0c03377, 2020, further permissions related to the material excerpted should be directed to the American Chemical Society. **b** Schematic showing the defect hypothesis for phospholipid coated hydrophobic MSNs (left). Cavitation onset peak negative pressures for hydrophobic MSNs coated with different lipids with different tail lengths (right). Adapted from reference [79], Copyright 2019, American Chemical Society

Following these pioneering studies, several GSNs have been developed using different materials, including mesoporous silica, porous silicon, gold, and polymers (Fig. 3) [34–36, 39–41, 44, 46–49, 70, 73, 78–87]. While, as discussed in the previous section, the exact mechanism of cavitation inception by GSNs is yet to be understood, these studies found that a hydrophobic surface or cavity is required to prepare GSNs with high acoustic activity (i.e., cavitation inception at low acoustic intensities). MSNs are the most commonly used type of particles to prepare GSNs. For gas stabilization, MSNs have been hydrophobically modified using alkyl- or fluoroalkyl silanes such as triethoxy silanes with octyl, dodecyl side chains, or silazanes such as hexamethyldisilazane (HMDS) [34, 36, 39, 78, 80]. In some studies, the hydrophobically modified MSNs were directly used [36, 44, 78], whereas others used amphiphilic molecules such as lipids, block copolymers, or β-cyclodextrin (β-CD) to disperse them in aqueous solutions [34, 39–41, 46]. Stabilizing the hydrophobic nanoparticles with an amphiphilic molecule can prevent their aggregation in biological solutions. In addition, the amphiphilic layer can be modified with functional molecules for specific applications such as targeted tumor ablation or molecular ultrasound imaging [80]. Recently, our group demonstrated that using an optimal amount of an amphiphilic polymer (Pluronic F127) and HMDS modified MSNs, GSNs with high acoustic activity could be prepared (Fig. 4a) [39]. We found that high F127 concentrations could cause the loss of acoustic activity, most likely due to the complete coverage of the nanoparticle surface with F127 molecules. Whereas at low F127 concentrations, particles showed high acoustic activity but did not form stable colloids in buffer solutions due to the low F127 surface coverage. This study speculated that at an optimal F127 concentration range, the nanoparticle surface was partially coated with F127 molecules where uncoated hydrophobic portions of the surface (i.e., defects) enable acoustic activity and polymer-coated portions provide stability in buffer solutions.

The previous observations by Blum et al. [79] also support the defect hypothesis (Fig. 4b). They stabilized hydrophobic MSNs using phospholipids with different lipid tail lengths. The authors found that the phase status of the lipid layer around the nanoparticle was crucial to prepare GSNs with high acoustic activity. The nanoparticles could initiate cavitation when the lipids were kept below their melting temperature in the gel phase. However, they lost their acoustic activity if the temperature was above the melting point of the stabilizing lipid layer. To explain these observations, the authors proposed that nanobubbles could be stabilized in the defects present on the gel-phase lipid layers.

Coating the hydrophobic nanoparticles with amphiphilic molecules can also improve the stability of their ultrasound responsiveness in biological solutions such as serum or whole blood. Adsorption of proteins and other biological molecules to the hydrophobic nanoparticle surfaces in biological fluids can reduce their acoustic activity. In fact, several studies reported a decrease in the acoustic activity of hydrophobic MSNs in the presence of albumin [36, 44, 78]. In comparison, better stability in biological solutions has been reported in the studies where nanoparticles were coated with an amphiphilic layer [34, 39].

**Fig. 5:**
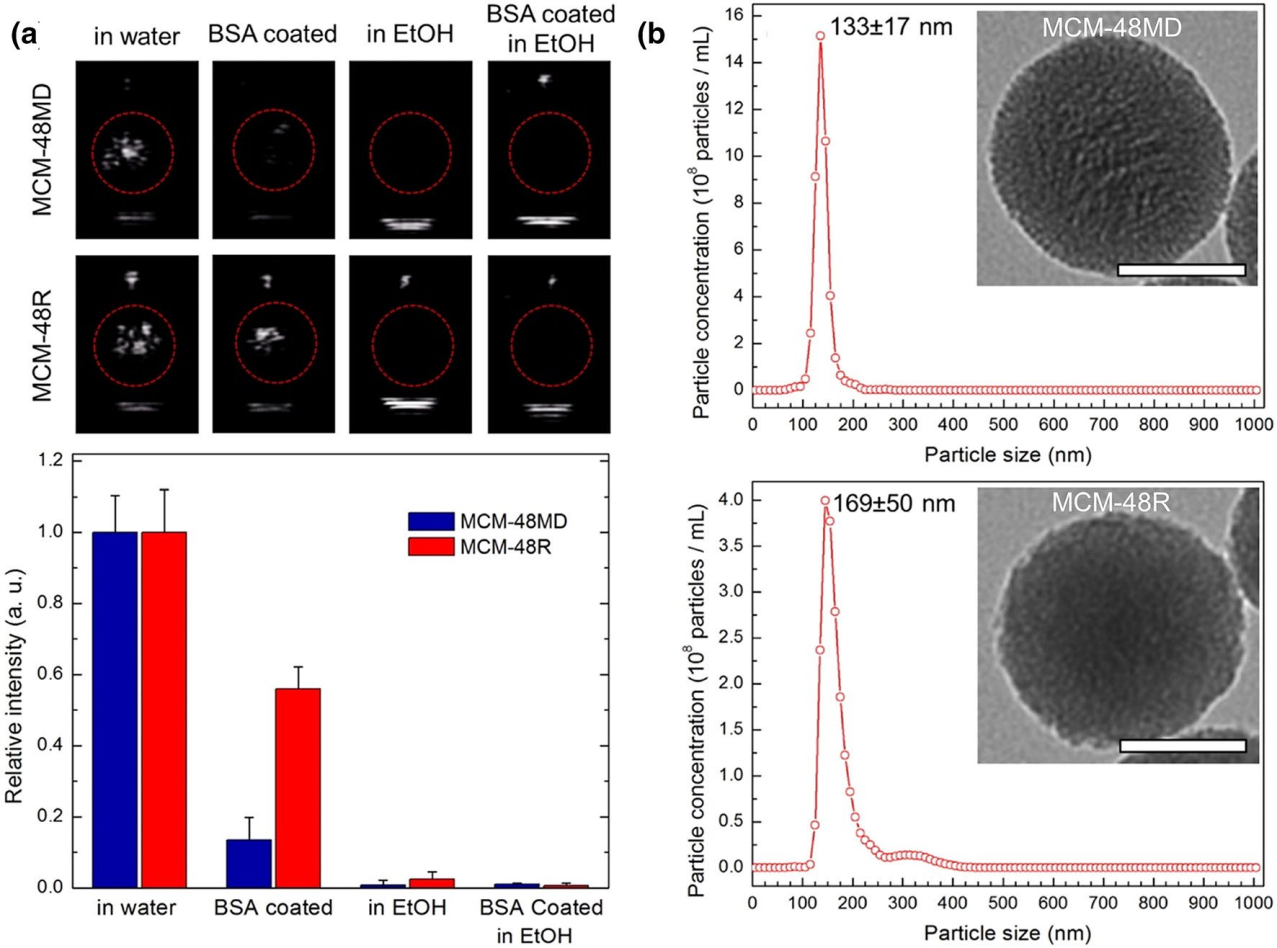
**a** Representative images showing the cavitation events as bright spots generated by MSN based GSNs under HIFU insonation of nanoparticles with or without BSA coating in ethanol (90%) or water (top). Calculated relative intensities from the acquired movies of nanoparticles during HIFU insonation (bottom). **b** Nanoparticle tracking analysis of the nanoparticles in water. Insets show the TEM images. Scale bars are 50 nm. Nanoparticles; MCM-48MD: hydrophobically modified MSNs using methyltrimethoxysilane, MCM-48R: MSNs with rough nanoparticle surfaces. Adapted from reference [78], Copyright 2016, American Chemical Society

While initial studies with MSNs accepted that gas stabilized inside the pores of the particles are forming micron-sized bubbles under reduced acoustic pressures, later studies showed that this is not necessarily the case. On this subject, Goodwin and coworkers [78] prepared a series of MSNs with different pore structures and surface morphologies. They investigated the acoustic activity of nanoparticles in the presence and absence of ethanol or albumin (Fig. 5). While ethanol can dissolve both the gas pockets stabilized inside the pores and surface of nanoparticles, albumin can only destabilize the ones at the particle surface as it cannot penetrate into the pores of MSNs due to its larger size (~ 5 nm) than the pore openings (2–3 nm). They found that incubating the particles with either ethanol or albumin could reduce the acoustic activity of nanoparticles. Thus, the authors concluded that acoustic cavitation was initiated by mostly surface gas pockets, and gas stabilized inside the pores has little or no contribution. A later study by Yeh and coworkers also reported similar observations with hydrophobically modified MSNs and solid silica nanoparticles [44]. Nevertheless, porosity appeared to be important to prepare MSNs with good acoustic activity [34, 44]. A previous study found that porous silica particles showed a much higher acoustic activity than solid silica nanoparticles with the same size and surface modification [34]. Thus, the authors speculated that nanoparticle porosity could improve the acoustic activity of nanoparticles by increasing the hydrophobicity of the nanoparticles and introducing ‘defects’ to the surface, both of which can help to improve the gas pocket adsorption and stabilization on the surface.

Beyond MSNs, several other nanoparticles with different morphologies have been used to prepare GSNs. Another common way in the literature is to use particles with large cavities, such as cup-shaped polymer or inorganic particles. Kwan et al., developed GSNs using divinylbenzene crosslinked poly(methyl methacrylate) (PMM) coated polystyrene (PS) nanocups [37]. Later similar GSNs have also been developed using different nanoparticles; gold nanocones, titanium dioxide nanocups, and multi-cavity poly lactic-co-glycolic acid (PLGA) particles [47, 81, 88]. These cup-shaped particles are believed to entrap gas pockets in their cavities, which in turn can generate micron-sized bubbles under ultrasound irradiation. Nevertheless, gas pocket stabilization on their hydrophobic outer surfaces is also possible.

Another interesting GSN example has been developed by Letho and coworkers using Janus porous silicon nanoparticles [35]. This study modified particle surfaces with polyethylene glycol (PEG) to provide dispersibility in aqueous solutions, and the pore walls were hydrophobically modified for gas stabilization. Similar to cup-shaped particles, gas stabilization in their large pores (> 10 nm) is possible for these nanoparticles. Other examples of GSNs include gold nanorods coated with hydrophobic peptides, zinc oxide nanoparticles, and Polytetrafluoroethylene (PTFE) nanoparticles [70, 83, 84].

## Applications of GSNs in ultrasound theranostics of cancer

### Ultrasound imaging

**Fig. 6:**
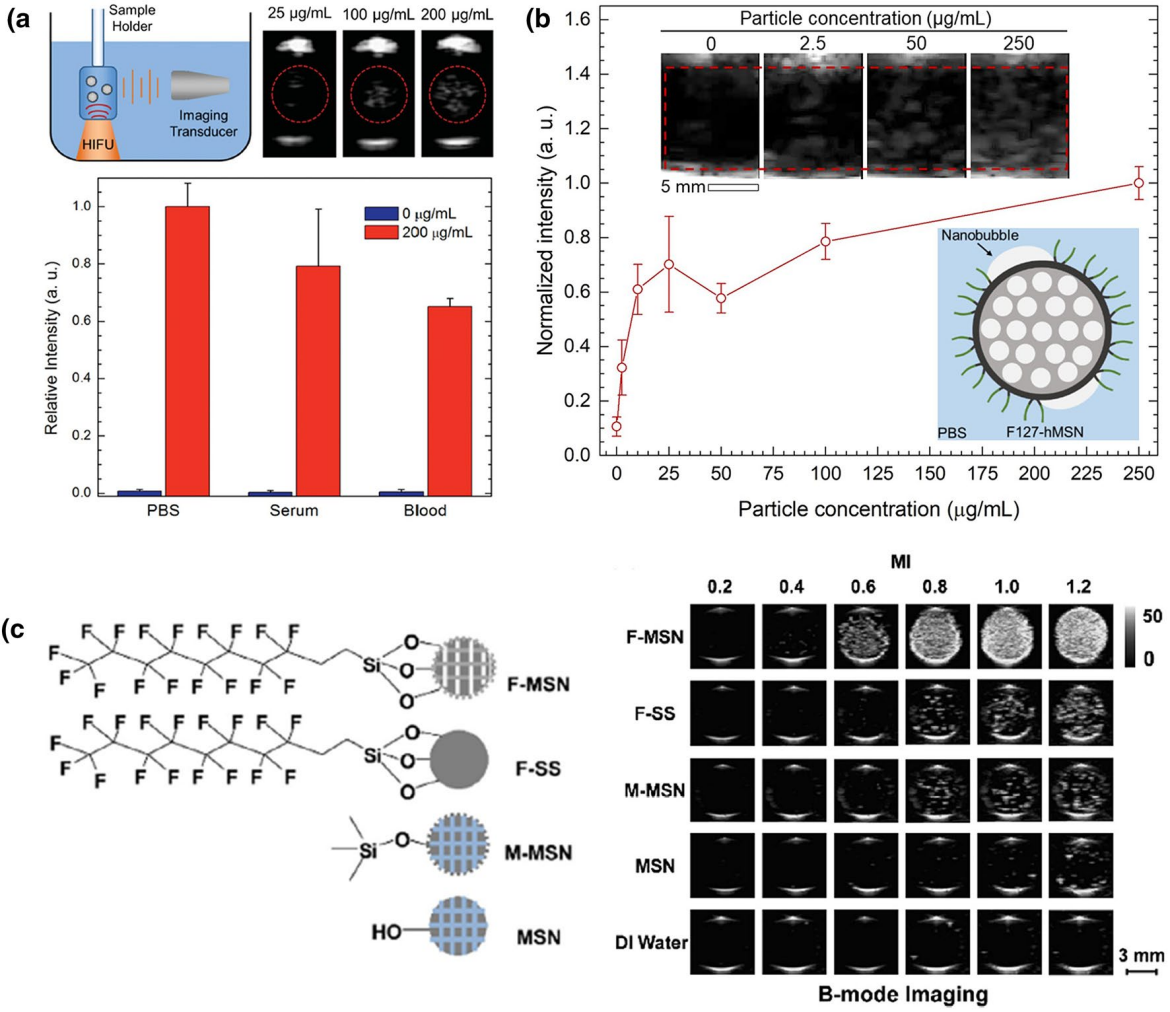
Ultrasound contrast generation by different MSN based GSNs. **a** Ultrasound contrast enhancement by F127 coated hydrophobic MSNs under HIIFU insonation in different media. Adapted from reference [34], Copyright 2016, John Wiley and Sons. **b** Average ultrasound intensity generated by F127 coated hydrophobic MSNs at different particle concentrations during ultrasound imaging at 2.5 MHz, and MI = 1.4. Adapted from reference [39], https://pubs.acs.org/doi/10.1021/acsomega.0c03377, 2020, further permissions related to the material excerpted should be directed to the American Chemical Society. **c** Ultrasound images of differently modified MSNs or solid silica nanoparticles at different MI values under B-mode imaging. Adapted from reference [36], Copyright 2017, Elsevier

Micron-sized bubbles generated by GSNs can be detected as bright spots in ultrasonograms using a conventional ultrasound imaging system as they strongly scatter ultrasound pulses. Thus, they can be applied for molecular ultrasound imaging of cancer. Unlike conventional ultrasound contrast agents such as microbubbles and sub-micron droplets, GSNs with small sizes (< 200 nm) can penetrate into the tumor tissue enabling imaging of molecular targets expressed there [89, 90]. To develop an ultrasound contrast agent based on GSNs, Goodwin and coworkers hydrophobically modified the surfaces of MSNs with octadecyl groups and stabilized the particles in aqueous media using an amphiphilic copolymer, Pluronic F127 (P-hMSN) [34]. F127 molecules can rapidly assemble on the surface of octadecyl modified MSNs through the hydrophobic interactions, and their hydrophilic (PEG) moieties provide dispersibility and colloidal stability in buffer solutions. The P-hMSNs could generate acoustic cavitation events in biological solutions, including serum and whole blood under HIFU insonation at a frequency of 1.1 MHz and a PNP of 9.87 MPa. Their results showed that the acoustic cavitation events generated by P-hMSNs could be imaged using harmonic ultrasound imaging (Fig. 6a). Importantly, under continuous HIFU insonation, ultrasound contrast generation could be achieved for at least 20 min. They also demonstrated that P-hMSN could be stored in PBS for at least 4 months or as lyophilized powders without any loss in their ultrasound responsiveness. Later the same authors prepared similar GSNs using PEGylated phospholipid monolayer stabilized dodecyl modified MSNs (PL-dMSN) [80]. They also modified the particles with folic acid (FA-PL-dMSN) to target them to cancer cells. Remarkably, they showed that the nanoparticles could remain acoustically active even after internalization into cancer cells and produce ultrasound contrast under 1.1 MHz HIFU insonation at a PNP of 10.6 MPa. With their small size and exceptional stability, such GSNs are very promising for molecular ultrasound imaging. However, a critical limitation of these GSNs is the requirement of HIFU activation pulses, which complicates the ultrasound imaging system and brings safety issues. In addition, the use of HIFU limits imaging volume to the volume of its focal zone, which is usually a few cubic millimeters [4]. Thus, to obtain images from larger volumes, the whole region of interest should be scanned with subsequent HIFU pulses, and the individual images should be compiled.

To address this limitation, our group has recently developed GSNs based on F127 stabilized HMDS modified MSNs (F127-hMSN) that can generate acoustic cavitation at low acoustic intensities, which can be delivered by using a medical imaging transducer (Fig. 6b) [39]. Thus, both activation of GSNs and imaging of the generated micron-sized bubbles can be performed using the same transducer. To improve the acoustic activity of particles, we carefully optimized the F127 coating step. Our studies showed that fine-tuning the F127 concentration was critical to obtain GSNs with good dispersibility in buffer solutions and high ultrasound responsiveness. Under optimized conditions, F127-hMSNs could generate ultrasound contrast at mechanical indices as low as 0.7, which is well below the FDA safety limit of 1.9. The cavitation events initiated by F127-hMSN could be continuously imaged for at least 20 min using B-mode ultrasound imaging (2.5 MHz plane wave pulses with PNPs of ~ 1–2.2 MPa) at particle concentrations as low as 2.5 µg/mL. To investigate if the cavitation events generated by F127-hMSNs could cause cellular damage, we used flow cytometry to detect dead or ablated cells. F127-hMSNs did not cause any detectable cell damage at imaging conditions indicating their potential safety for ultrasound imaging applications. In addition, it was found that F127-hMSN partially degraded in simulated body fluids at 37 °C. Interestingly, after 1 week of incubation, only the inner parts of the particles were dissolved, and the outer surface remained intact even after 4 weeks of incubation. While further studies are needed to understand the degradation behavior of F127-hMSNs better, these results suggested potential biodegradation in in vivo conditions.

**Fig. 7:**
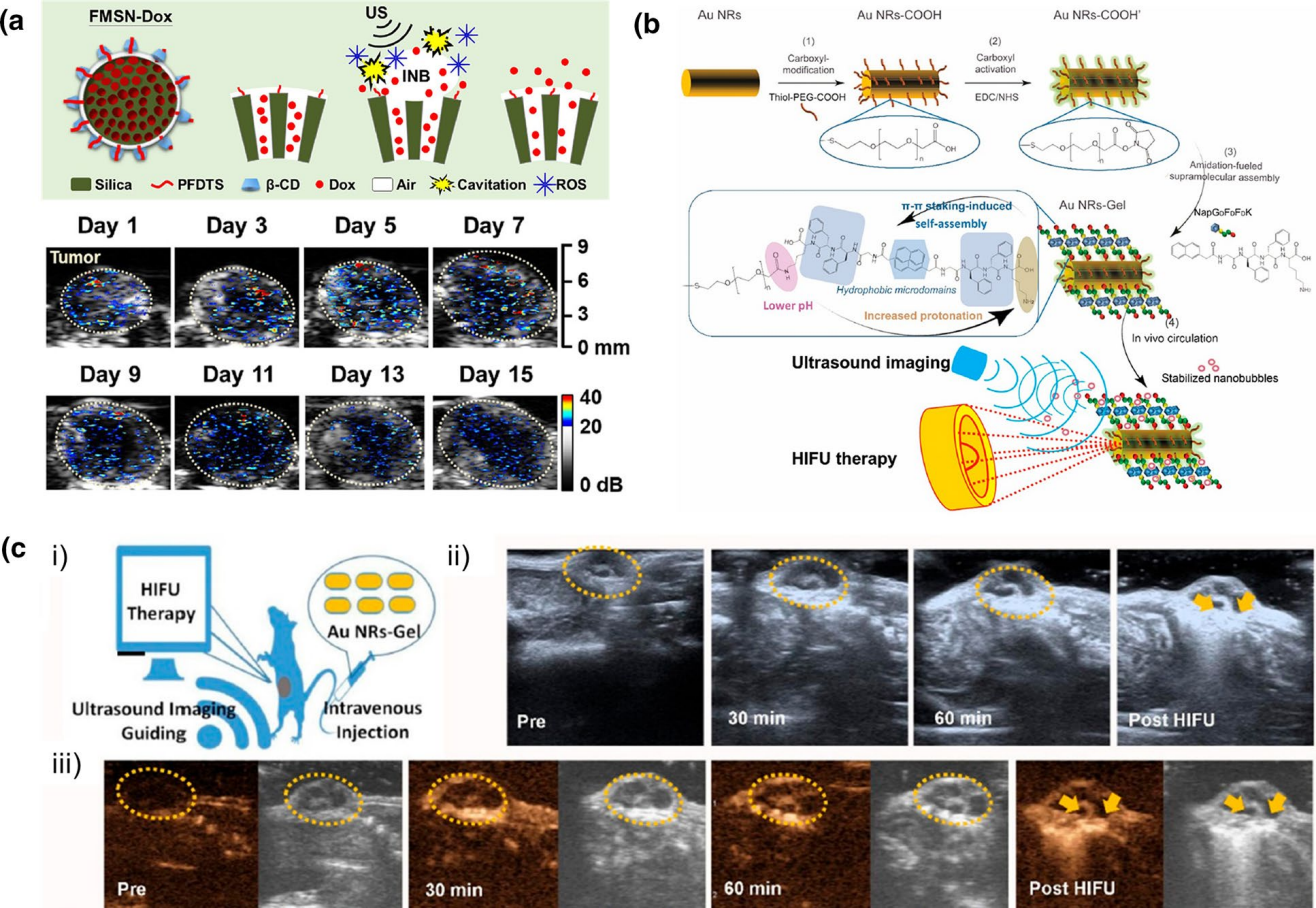
Examples of GSNs used for in vivo ultrasound imaging. **a** Schematic showing drug loaded hydrophobic MSNs coated with β-CD (above) and in vivo ultrasound contrast enhancement as a result of cavitation generation by GSNs over 15 days. Adapted from reference [40], Copyright 2020, Elsevier. **b** Schematic showing the preparation of peptide hydrogel coated gold nanorods and nanobubble stabilization by them. **c** Ultrasound contrast enhancement by intravenously injected peptide hydrogel coated gold nanorods at different time points after nanoparticle injection and after HIFU treatment. Adapted from reference [84], Copyright 2019, American Chemical Society

Yeh and coworkers have also developed GSNs using hydrophobically modified MSNs [36]. The particles produced strong contrast enhancement during B-mode imaging using a transducer operating at 7.5 MHz at mechanical indices as low as 0.6 (Fig. 6c). As the particles developed in this study were directly used without amphiphilic stabilization, they demonstrated poor stability in 50% serum with more than a twofold reduction in the ultrasound contrast intensity. More recently, the same group has prepared β-CD stabilized hydrophobic MSNs [40]. They demonstrated that intravenously injected particles could accumulate in the transgenic adenocarcinoma of the mouse prostate (TRAMP) tumor model and be imaged using an ultrasound imaging transducer with a frequency of 15 MHz. Importantly, intravenously injected particles showed excellent in vivo signal stability and enabled ultrasound contrast enhancement even 15 days after particle injection (Fig. 7a).

Lehto and coworkers developed a distinct GSN using anisotropically modified porous silicon nanoparticles (J-Psi) [35]. These J-Psi NPs were prepared using a nanostoper technique that enables PEGylation of the external surface without modifying the hydrophobic pore walls. The hydrophobic pores of the particles enabled gas stabilization, and their PEGylated surfaces ensured stability in biological fluids. The theoretical calculations found that J-Psi could nucleate bubbles with sizes around 1.1 µm. The authors showed that the micron-sized bubbles generated by J-Psi could be imaged in vitro using B-mode ultrasound imaging at a mechanical index of 1.4. One limitation of this study is the challenge in the large-scale production of porous silicon nanoparticles with uniform shapes and narrow size distribution. However, the site-specific surface modification method reported in this study could potentially be extended to prepare similar GSNs using different nanoparticles such as large pore MSNs or gold nanocages [91, 92].

Another type of GSN for ultrasound imaging has been developed by Wang and coworkers using a hydrophobic peptide (NapG_D_F_D_F_D_K) and gold nanorods (AuNRs-Gel) (Fig. 7b) [84]. The authors formed an amphiphilic hydrogel layer around the particles with a hydrophobic interface and a water-rich surface layer to realize gas stabilization and dispersibility in aqueous solutions. Importantly, they demonstrated that the AuNRs-Gel could be used as contrast agents for in vivo ultrasound imaging (Fig. 7c). They observed a 2–threefold enhancement in the tumor ultrasound contrast 30 min after intravenous injection of AuNRs-Gel in both B-mode and contrast-enhanced ultrasound images obtained using an 18 MHz linear-array transducer. Later the authors extended this method to develop similar GSNs based on MSNs [85].

### Mechanical tumor ablation

HIFU has been applied to treat several different solid malignancies in recent years, including prostate, breast, brain, pancreas, and ovarian cancers [4, 93–95]. Currently, some commercial HIFU devices are in use in several clinical trials worldwide. These devices typically apply millisecond long pulses with PNPs of ~ 1–5 MPa to generate a rapid temperature increase in the focal zone and induce coagulative necrosis to ‘thermally’ ablate the treated tissue. However, incomplete tumor ablation due to the heat sink effect of flowing blood or insufficient temperature increase in the peripheral areas often leads to tumor recurrence [96, 97]. Alternatively, microsecond range pulses with higher PNP (typically > 20 MPa) could be used to generate a strong cavitation activity that results in the ‘mechanical’ disintegration of the treated tissue [98]. Mechanical ablation, or histotripsy, can potentially provide a more complete tumor ablation than thermal HIFU treatment [99–101]. However, the high acoustic pressures required to generate a strong and durable cavitation activity make it challenging to safely utilize this method in the clinic [102].

**Fig. 8:**
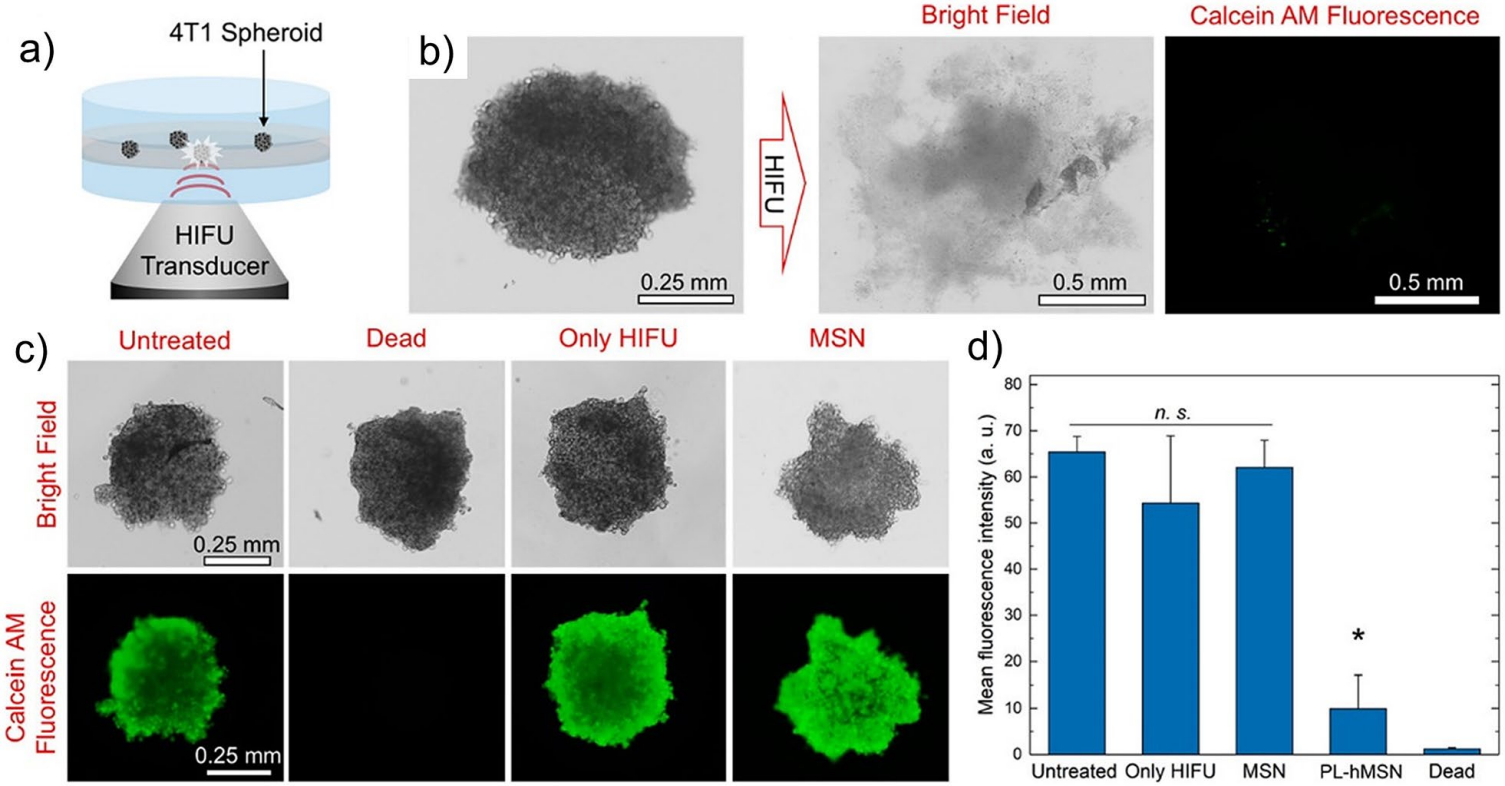
Ablation of tumor spheroids using HIFU and GSNs. **a** Schematic of the experimental setup. **b** Bright field and fluorescence images of a Calcein AM stained spheroid before and after HIFU treatment. **c** Representative bright field and fluorescence images of Calcein AM stained spheroids at different conditions: an untreated, a heat treated (dead), a HIFU treated in the absence of GSNs, and HIFU treated in the presence of nonresponsive MSNs. **d** Mean fluorescence intensities for spheroids treated at different conditions. Adapted from reference [46], Copyright 2018, American Chemical Society

GSNs can lower the cavitation threshold and enable high cavitation activity at safer pressure levels. For example, Goodwin and coworkers have used phospholipid stabilized hydrophobic MSNs (P-hMSNs) to achieve mechanical cell ablation at low acoustic intensities. They demonstrated that in the presence of a low amount of P-hMSN (10–100 μg mL^−1^), red blood cells and tumor spheroids encapsulated in tissue-mimicking agarose gels could be ablated using relatively low intensity focused ultrasound (1.1 MHz) treatment at a PNP of ~ 11 MPa and duty cycle of only ~ 0.01% (Fig. 8) [46]. They found that in the presence of P-hMSN, tumor spheroids could be almost completely disintegrated with a ~ 85% decrease in cellular viability. On the other hand, there was no detectable change in the cellular viability and spheroid morphology in the absence of P-hMSN. Also, they did not detect any temperature increase in the tissue-mimicking gels after ultrasound treatment indicating that cell ablation was purely mechanical. Similarly, using F127-coated hydrophobic MSNs, our group showed that cancer cells could be effectively ablated at low acoustic intensities where a temperature increase of less than 1 °C was detected after ultrasound treatment [39]. Goodwin and coworkers have also prepared targeted GSNs by modifying the surfaces of PL-hMSN with folic acid [80]. They demonstrated that the folic acid-modified GSNs could be uptaken by MDA-MB-231 breast cancer cells without showing any significant cytotoxicity at particle concentrations up to 200 µg/mL. They found that nanoparticles internalized into cancer cells could be used to ablate the cells when exposed to focused ultrasound (1.1 MHz) pulses with a PNP of ~ 11 MPa and a duty cycle of ~ 0.5%.

**Fig. 9:**
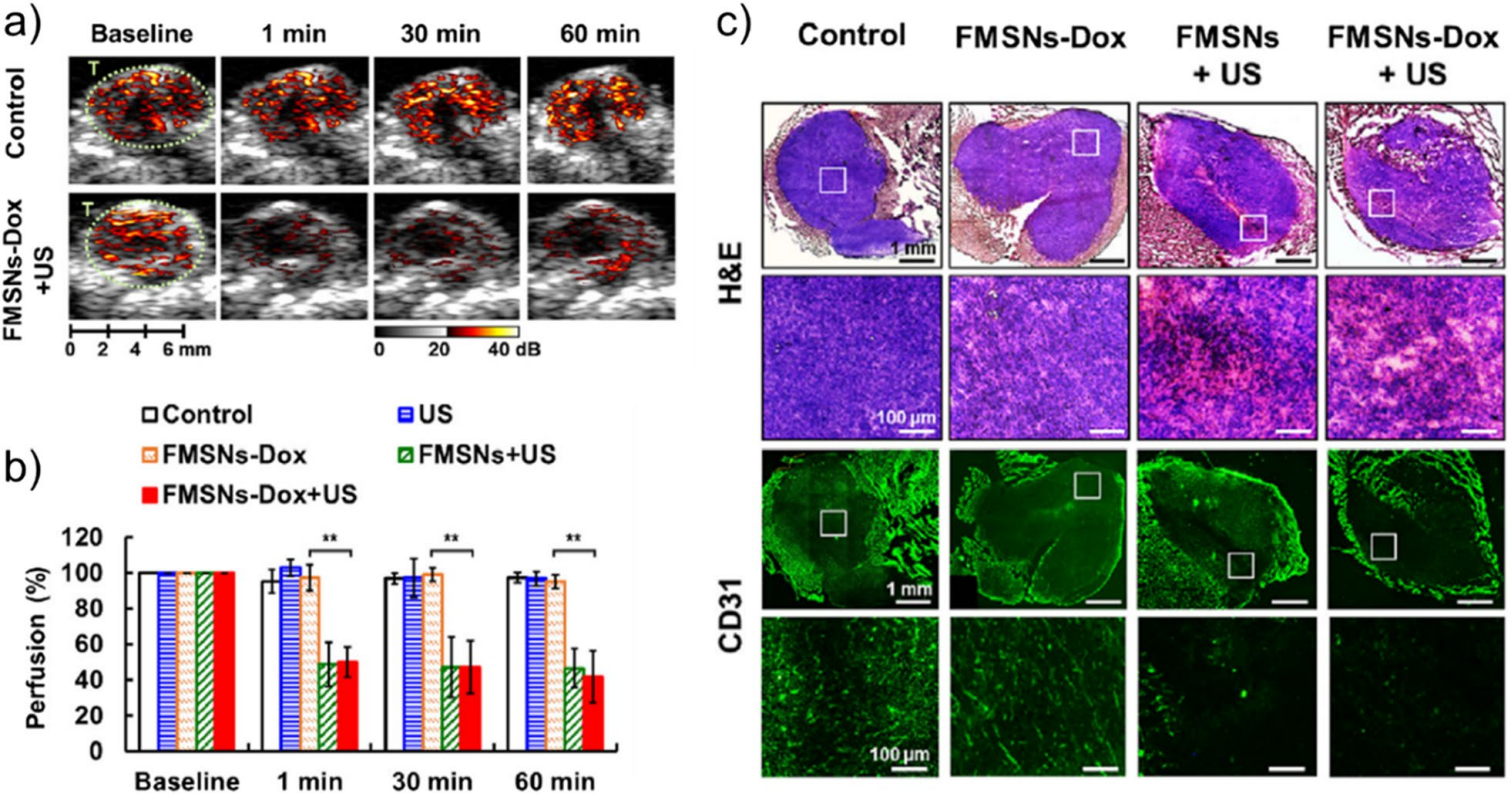
In vivo tumor ablation using GSNs. **a** Ultrasound images showing tumor perfusion of intravenously injected microbubbles showing anti-vascular effect of HIFU treatment in the presence of GSNs. **b** Quantification of tumor perfusion in mice treated with different conditions. **c** Histological analysis of tumors untreated or treated using different conditions. H&E staining shows the necrosis induced by GSNs and ultrasound. CD31 staining was used to visualize tumor vasculature. Adapted from reference [40], Copyright 2020, Elsevier

In vivo tumor ablation using GSNs has also been demonstrated. Yeh and coworkers prepared β-CD coated hydrophobic MSNs and used them for tumor vasculature disruption (Fig. 9) [40]. They observed anti-vascular effects in the presence of retro-orbitally injected nanoparticles and 2.5 ms long HIFU (2 MHz) pulses with a PNP of 5 MPa. Wang and coworkers used gold nanorods coated with supramolecular peptide hydrogels (AuNRs-Gel) to ablate HeLa xenografts in mice [84]. In addition, they utilized the ultrasound contrast generated by AuNRs-Gel for image guidance during HIFU treatment. They showed that upon intravenous injection of the GSNs, the average intensity of both B-mode and contrast mode images in the tumor region quickly increased in the first hour after injection. Terminal deoxynucleotidyl transferase dUTP nick end labeling (TUNEL) and hematoxylin and eosin (H&E) staining on the treated tumor slices showed significant cell destruction, liquefaction necrosis, and apoptosis. Later, the same authors also obtained similar results using supramolecular peptide hydrogel-coated MSNs [85].

### Localized drug delivery

**Fig. 10:**
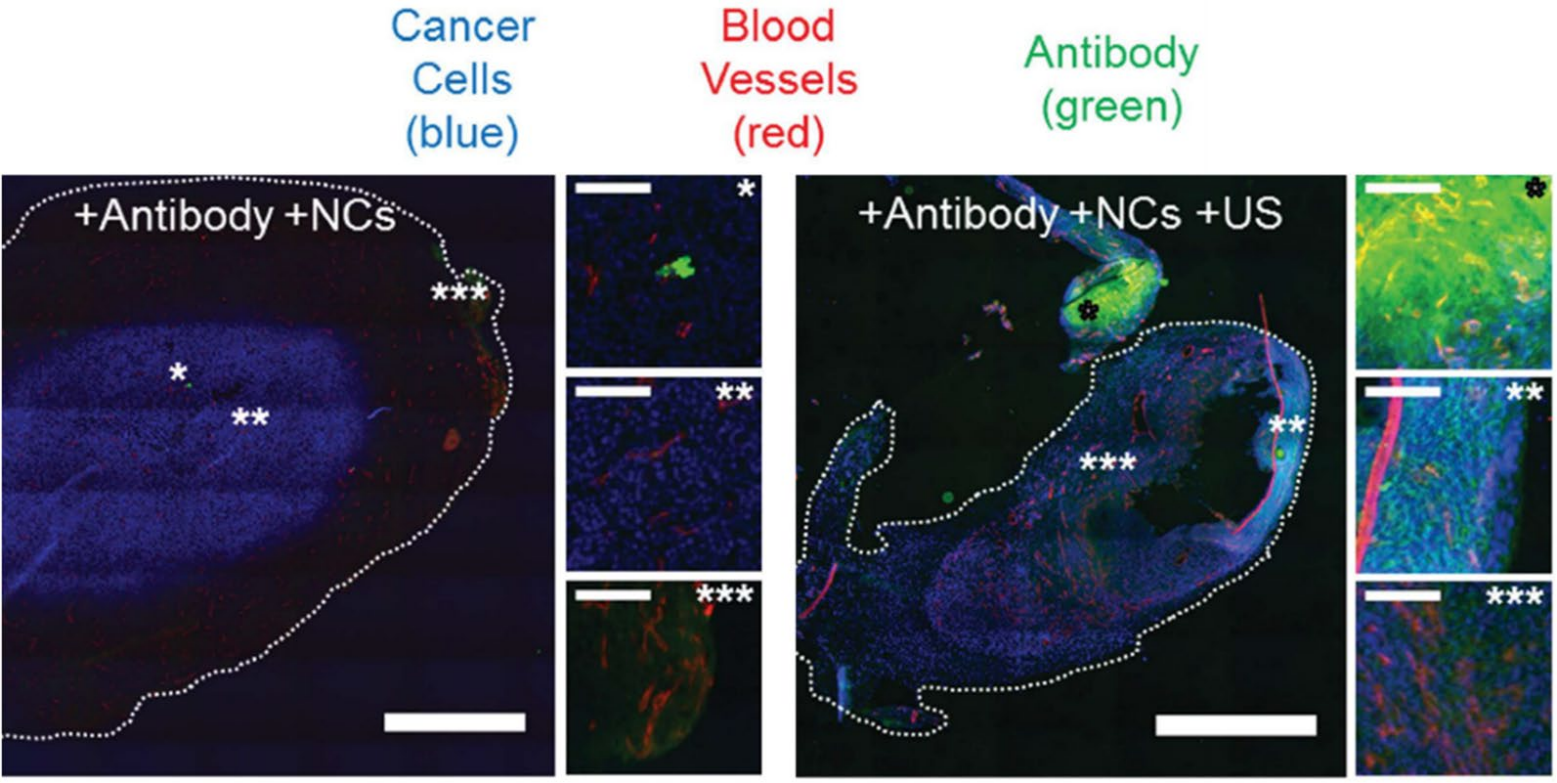
Representative fluorescence images showing antibody delivery into CT-26 tumors with polymer nanocups and ultrasound exposure. Scale bars are 1 mm in larger images and 0.1 mm in smaller images. Adapted from reference [37], Copyright 2015, John Wiley and Sons

GSNs have also been used for localized drug delivery applications using external ultrasound pulses. For example, Coussios and coworkers have developed GSNs based on divinylbenzene-crosslinked PMM coated PS nanocups and applied them to improve the tumor accumulation of a model therapeutic (Immunoglobulin G; IgG, mouse antibody) [37]. They demonstrated that cavitation events generated by intravenously injected nanocups could provide deep tumor penetration of freely circulating IgG molecules in a CT-26 colon cancer mouse model under ultrasound insonation (2 MHz center frequency and at 4.5 MPa) (Fig. 10). Later, polymer nanocups have also been used to improve tissue penetration of a therapeutic antibody (Cetuximab), a model vaccine (ovalbumin) and an oncolytic virus [43, 103, 104]. Remarkably, ex vivo studies with ovalbumin showed that in the presence of nanocups, ovalbumin could penetrate into porcine skin up to a distance of 500 µm when exposed to 10 ms long focused ultrasound pulses at a frequency of 256 kHz with PNPs < 1.5 MPa and 10% duty cycle [103]. Another significant result obtained in these studies was the demonstration of three to four-fold enhancement in the gene expression from vaccinia virus in the presence of nanocup induced cavitation [104]. In addition, the authors also compared the cavitation activity of nanocups with a microbubble cavitation agent, SonoVue, and observed ~ 10 times higher gene expression with nanocups [42]. This novel type of GSNs has already been commercialized by OxSonics Therapeutics with the brand name of SonoTran.

The cavitation events generated by GSNs could potentially diminish the efficacy of therapeutic molecules. For instance, mechanical effects induced during cavitation can denature or aggregate proteins or destroy viruses. Chemical cavitation effects such as ROS generation, on the other hand, could break chemical bonds or oxidize molecules. Thus, to understand if the cavitation events generated by nanocups can decrease the effectiveness of therapeutics, Myers et al. [105] treated a variety of small molecules, macromolecules, and viruses, including doxorubicin, cetuximab, adenovirus, and enveloped viral vectors with unfocused 0.5 MHz ultrasound pulses with PNP of 1.5 MPa in the presence of nanocups. Their results did not show any detectable decrease in the efficacy of any of the tested therapeutics when exposed to cavitation. Nevertheless, further in vitro and in vivo studies are needed to fully evaluate the cavitation effect on therapeutic molecules.

**Fig. 11:**
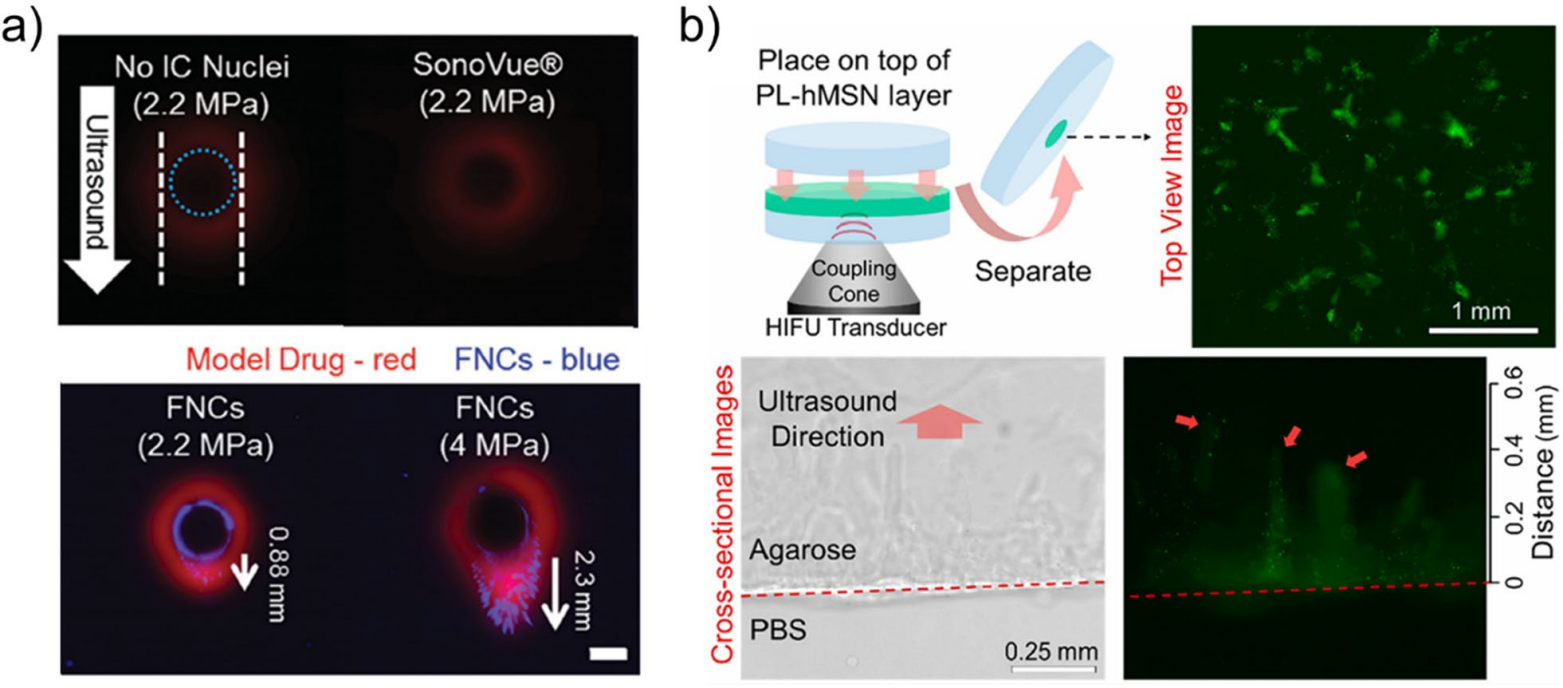
**a** Representative fluorescence images showing nanocup penetration to a tissue model hydrogel after ultrasound treatment. Adapted from reference [37], Copyright 2015, John Wiley and Sons. **b** Phospholipid stabilized hydrophobic MSN penetration to agarose gel due to the generated cavitation events. Nanoparticles were labeled with fluorescein for imaging. Adapted from reference [46], Copyright 2018, American Chemical Society

GSNs have also been used to improve the penetration of nanoparticles into tissue-mimicking gels or solid tumors. Coussios and coworkers have found that nanocups themselves could penetrate deep into tissue-mimicking hydrogels up to ~ 2 mm (Fig. 11a) or porcine skin up to ~ 700 µm under ultrasound insonation [37, 103]. Goodwin and coworkers have also found that phospholipid stabilized hydrophobic MSNs could penetrate into tissue-mimicking hydrogels up to a distance of ~ 500 µm when exposed to focused ultrasound (1.1 MHz) pulses with a PNP of 16.4 MPa and duty cycle of 0.017% (Fig. 11b) [46]. Nanocups have also been used to improve tissue penetration of other nanoparticles. Paris et al. [106] mixed nanocups with MSN suspensions and loaded them into a microchannel made of agarose. Under focused ultrasound (0.5 or 1.6 MHz with PNPs of 1–4 MPa) insonation (5% duty cycle), they observed that MSNs could be penetrated into agarose up to a distance of ~ 1 mm. Later, similar results were also obtained using gold nanocone-based GSNs [47].

Finally, Yeh and coworkers used doxorubicin-loaded MSN-based GSNs for enhanced drug delivery into TRAMP prostate tumors in mice [40]. They showed that cavitation generated by MSNs could disturb the tumor vasculature and improve the accumulation of doxorubicin-loaded particles in the tumor tissue. This study is an excellent example of how GSNs could be used for multimodal therapy of cancer. The cavitation events induced by GSNs could mechanically destroy the tumor tissue and improve the drug delivery at the same time.

### Sonodynamic therapy

Acoustic cavitation events generated by GSNs can produce ROS, and thus, can induce apoptotic cell death [107–109]. In addition, acoustic cavitation can activate sonosensitizers such as porphyrin dyes or titanium oxide nanoparticles. Therefore, GSNs can be administrated either solely or in combination with a sonosensitizer to improve the outcomes of sonodynamic therapy.

**Fig.12:**
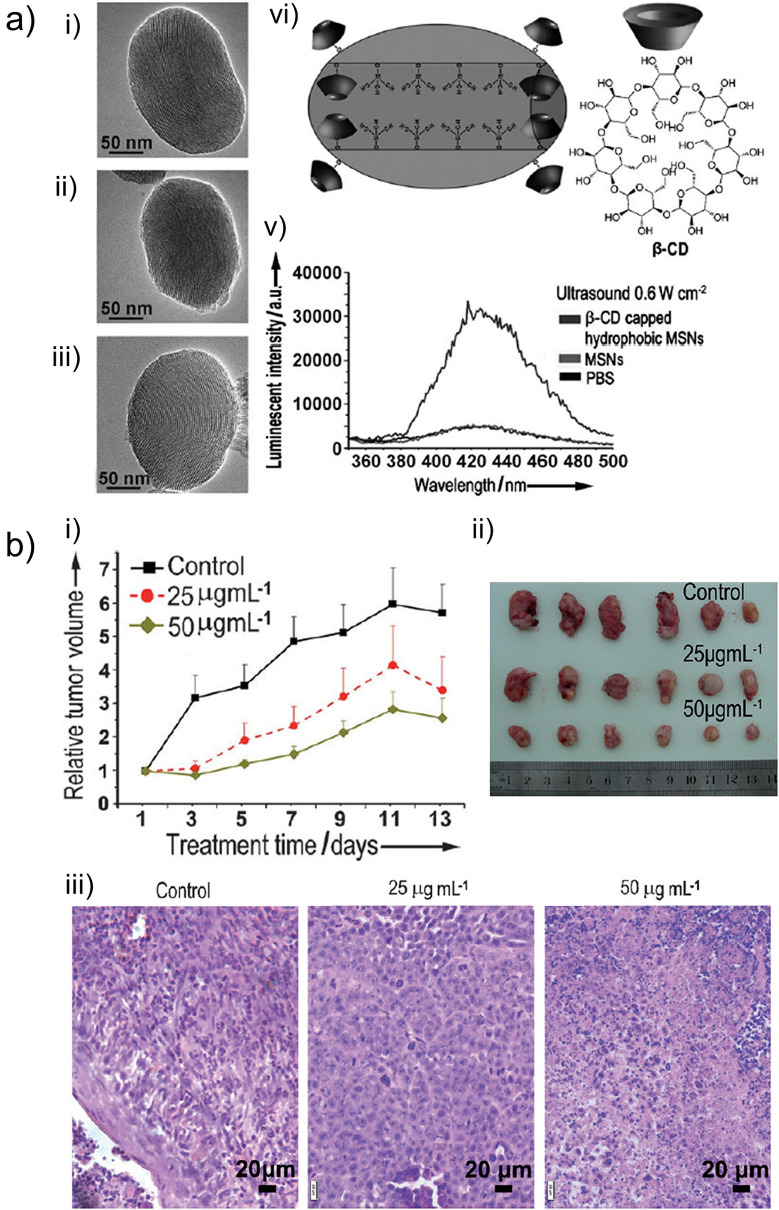
**a** TEM images of MSNs (i), hydrophobically modified MSNs (ii), and β-CD stabilized hydrophobic MSNs (iii). Schematic showing the molecular structure of hydrophobically modified MSN pore walls and β-CD (iv). Fluorescence assay showing in vitro ROS generation by MSNs after ultrasound exposure (v). **b** In vivo sonodynamic therapy using β-CD stabilized hydrophobic MSNs in ZR75-30 tumor model in mice. Relative tumor volumes of mice untreated or treated with ultrasound in the presence of nanoparticles at different concentrations (i). Photographs (ii) and H&E staining (iii) of tumors at end of the experiment. Adapted from reference [48], Copyright 2014, John Wiley and Sons

Wang and coworkers prepared HMDS modified MSNs (HMSN) and capped them with β-CD [48]. They showed that these particles could generate ROS in buffer solutions under low-intensity ultrasound irradiation (1 MHz) (Fig. 12a). They also showed that β-CD capped HMSN could effectively eliminate breast cancer ZR75-30 cells at low particle concentrations down to 5 µg/mL and at an acoustic intensity of 0.6 W/cm^2^. Finally, the authors used β-CD capped HMSNs to slow down the growth of ZR75-30 xenografts in mice under ultrasound treatment (0.8 W/cm^2^), which was applied every other day for 2 weeks (Fig. 12b). While the β-CD capped HMSNs were directly injected into the tumor in this study, the amount of injected nanoparticles was remarkably low; 250–500 ng, suggesting the high ultrasound responsiveness of β-CD capped HMSNs. Later the same authors also used hollow MSNs for sonodynamic therapy and validated them in in vitro experiments using ZR75-30 breast cancer cells [49]. Yeh and coworkers [40] also demonstrated ROS generation by intravenously injected β-CD capped hydrophobic MSNs under HIFU sonication in TRAMP prostate cancer model. They also combined sonodynamic therapy with chemotherapy and anti-vascular therapy to develop a more efficient multimodal therapy.

Kwan and coworkers [88] prepared GSNs based on titanium dioxide nanocups as both a cavitation agent and a sonosensitizer. They found that cavitation events could significantly improve the ROS generation capability of titanium dioxide nanoparticles under ultrasound sonication (33% duty cycle) using a focused ultrasound transducer with a frequency of 0.5 or 1.1 MHz and PNPs of 0.1–8 MPa. In in vitro studies, the authors demonstrated that in the presence of titanium dioxide-based GSNs, a methylene blue degradation of almost 3 orders of magnitude faster than previously developed titanium dioxide nanoparticle-based methods could be achieved.

## Conclusions and future outlook

In summary, recently developed robust and small-sized GSNs have an excellent potential in cancer imaging and therapy. In the last decade, we have witnessed rapid growth in the field with the development of several different GSNs. These nanoparticles have already been utilized in different applications to detect and treat cancer [40, 48, 80, 104] and others such as cardiovascular diseases [73, 79, 87]. These studies have also significantly increased our understanding of the cavitation inception by nanoparticles. Nevertheless, before their clinical transition, more studies should be performed to address some preclinical questions.

First of all, there is still very little known about their biocompatibility and biodegradability. The hydrophobic coatings of GSNs can potentially slow down or avoid their degradation by preventing water penetration into particle networks even if particles with known biodegradability characteristics, such as MSNs or porous silicon nanoparticles, are used to prepare GSNs. In fact, our previous study showed that F127 stabilized hydrophobic MSNs only partially degraded in simulated body fluid after 4 weeks of incubation [39]. At the same experimental conditions, we found that MSNs without any hydrophobic modification wholly degraded in a few days (unpublished results). Yeh and coworkers also observed similar degradation kinetics for β-CD stabilized hydrophobic MSNs [40]. Nevertheless, Kwan and coworkers [81] prepared degradable gas stabilizing microparticles using multi-cavity PLGA particles, suggesting that it may also be possible to prepare biodegradable GSNs with high ultrasound responsiveness by optimizing the GSN manufacturing steps. Also, detailed long-term in vivo studies should be performed to evaluate the potential toxicity and immunogenicity of GSNs and their degradation products.

Regarding safety, another challenge in the field is to generate cavitation in the target tissue without damaging the healthy tissue lying on the path of ultrasound pulses [110]. This is especially important for ultrasound imaging applications where strict safety measures are taken for clinical translation [111]. Some in vitro studies found undetectable cell death by the cavitation events generated by GSNs under imaging conditions where low-intensity ultrasound was used [34, 39]. While these studies suggest the potential safety of GSNs for ultrasound imaging, the conditions used in the in vitro cell environment are very different from the actual tissue microenvironment. Thus, in vivo studies are needed to evaluate their safety in a more realistic model.

Our knowledge regarding the stability of ultrasound responsiveness of GSNs and their pharmacokinetics in in vivo conditions is also limited. There has not been any study yet that systemically examines these issues. However, the preliminary results obtained by Yeh and coworkers [40] are promising, where the authors showed that intravenously injected GSNs could accumulate in tumors and remain acoustically active there for at least 15 days after injection. This result suggests that GSNs could be repeatedly used to generate cavitation events in the tissue at different time points after a single dose injection. Thus, the GSN dose needed for a therapy or imaging application could be reduced, improving safety by reducing the potential side effects. Our group also found that PEGylated GSNs could be found in the circulation even 2 days after intravenous injection, and they could effectively accumulate in a syngenic mouse tumor model (unpublished results).

As discussed above, GSNs have been applied for different applications, including drug delivery, ultrasound imaging, tumor ablation, and sonodynamic therapy. However, there are many other potential applications yet to be explored. Their nanoscale size makes them suitable for molecular ultrasound imaging of biomarkers expressed in the tumor tissue, which are not accessible to microbubbles. While one study validated GSNs for molecular ultrasound imaging in in vitro [80], this has not been demonstrated in vivo yet. Another potential application is in immunotherapy [10]. It is well known that ablation of cancer cells by ultrasound can induce innate and adaptive immune responses as a result of released tumor antigens (TAs) and danger-associated molecular patterns (DAMPs) [112]. GSNs could be applied to induce cavitation events in the tumors at lower acoustic intensities to more efficiently and safely release TAs and DAMPs and may generate a more robust immune response. We believe that these applications and many others of GSNs will be demonstrated in the near future.

## Data Availability

Any data related to this review are available from the corresponding author on request.

## Abbreviations

HIFU: High intensity focused ultrasound
FDA: The United States Food and Drug Administration
EPR: Enhanced permeability and retention
GSNs: Gas-stabilizing nanoparticles
PNP: Peak negative pressure
ROS: Reactive oxygen species
AFM: Atomic force microscopy
TIRF: Total internal reflection fluorescence
MSNs: Mesoporous silica nanoparticles
Cryo-EM: Cryogenic electron microscopy
NTA: Nanoparticle tracking analysis
HMDS: Hexamethyldisilazene
β-CD: β-Cyclodextrin
PMM: Poly(methyl methacrylate)
PS: Polystyrene
PLGA: Poly lactic-co-glycolic acid
PEG: Polyethylene glycol
PTFE: Polytetrafluoroethylene
TRAMP: Transgenic adenocarcinoma of the mouse prostate
TUNEL: Terminal deoxynucleotidyl transferase dUTP nick end labeling
H&E: Hematoxylin and eosin
IgG: Immunoglobulin G
TAs: Tumor antigens
DAMPs: Danger-associated molecular patterns (DAMPs

## References

[1] Kiessling F, Fokong S, Bzyl J, Lederle W, Palmowski M, Lammers T (2014). Recent advances in molecular, multimodal and theranostic ultrasound imaging. Adv. Drug Deliv. Rev..

[2] Rix A, Lederle W, Theek B, Lammers T, Moonen C, Schmitz G, Kiessling F (2018). Advanced ultrasound technologies for diagnosis and therapy. J. Nucl. Med..

[3] James ML, Gambhir SS (2012). A molecular imaging primer: modalities, imaging agents, and applications. Physiol. Rev..

[4] Kennedy JE (2005). High-intensity focused ultrasound in the treatment of solid tumours. Nat. Rev. Cancer.

[5] ter Haar G, Coussios C (2007). High intensity focused ultrasound: physical principles and devices. Int. J. Hyperth..

[6] Babalola O, Lee T-HJ, Viviano CJ (2018). Prostate ablation using high intensity focused ultrasound: a literature review of the potential role for patient preference information. J. Urol..

[7] Ahmed M, Solbiati L, Brace CL, Breen DJ, Callstrom MR, Charboneau JW, Chen M-H, Choi BI, de Baère T, Dodd GD, Dupuy DE, Gervais DA, Gianfelice D, Gillams AR, Lee FT, Leen E, Lencioni R, Littrup PJ, Livraghi T, Lu DS, McGahan JP, Meloni MF, Nikolic B, Pereira PL, Liang P, Rhim H, Rose SC, Salem R, Sofocleous CT, Solomon SB, Soulen MC, Tanaka M, Vogl TJ, Wood BJ, Goldberg SN (2014). Image-guided tumor ablation: standardization of terminology and reporting criteria—a 10-year update. Radiology.

[8] Mitragotri S (2005). Healing sound: the use of ultrasound in drug delivery and other therapeutic applications. Nat. Rev. Drug Discov..

[9] Lentacker I, De Cock I, Deckers R, De Smedt SC, Moonen CTW (2014). Understanding ultrasound induced sonoporation: definitions and underlying mechanisms. Adv. Drug Deliv. Rev..

[10] Unga J, Hashida M (2014). Ultrasound induced cancer immunotherapy. Adv. Drug Deliv. Rev..

[11] Hynynen K (2008). Ultrasound for drug and gene delivery to the brain. Adv. Drug Deliv. Rev..

[12] Timbie KF, Mead BP, Price RJ (2015). Drug and gene delivery across the blood-brain barrier with focused ultrasound. J. Control. Release.

[13] Yildirim A, Blum NT, Goodwin AP (2019). Colloids, nanoparticles, and materials for imaging, delivery, ablation, and theranostics by focused ultrasound (FUS). Theranostics.

[14] Schutt EG, Klein DH, Mattrey RM, Riess JG (2003). Injectable microbubbles as contrast agents for diagnostic ultrasound imaging: the key role of perfluorochemicals. Angew. Chemie Int. Ed..

[15] Correas JM, Bridal L, Lesavre A, Méjean A, Claudon M, Hélénon O (2001). Ultrasound contrast agents: properties, principles of action, tolerance, and artifacts. Eur. Radiol..

[16] Miller DL, Averkiou MA, Brayman AA, Everbach EC, Holland CK, Wible JH, Wu J (2008). Bioeffects considerations for diagnostic ultrasound contrast agents. J Ultrasound Med.

[17] Stride E, Saffari N (2003). Microbubble ultrasound contrast agents: a review. Proc. Inst. Mech. Eng. Part. H. J. Eng. Med..

[18] Schneider M (1999). Characteristics of SonoVue^TM^. Echocardiography.

[19] Ferrara K, Pollard R, Borden M (2007). Ultrasound microbubble contrast agents: fundamentals and application to gene and drug delivery. Annu. Rev. Biomed. Eng..

[20] Zhou Y, Han X, Jing X, Chen Y (2017). Construction of silica-based micro/nanoplatforms for ultrasound theranostic biomedicine. Adv. Healthc. Mater..

[21] Garg S, Thomas AA, Borden MA (2013). The effect of lipid monolayer in-plane rigidity on in vivo microbubble circulation persistence. Biomaterials.

[22] Sindhwani S, Syed AM, Ngai J, Kingston BR, Maiorino L, Rothschild J, MacMillan P, Zhang Y, Rajesh NU, Hoang T, Wu JLY, Wilhelm S, Zilman A, Gadde S, Sulaiman A, Ouyang B, Lin Z, Wang L, Egeblad M, Chan WCW (2020). The entry of nanoparticles into solid tumours. Nat. Mater..

[23] Pandit S, Dutta D, Nie S (2020). Active transcytosis and new opportunities for cancer nanomedicine. Nat. Mater..

[24] Jain RK, Stylianopoulos T (2010). Delivering nanomedicine to solid tumors. Nat. Rev. Clin. Oncol..

[25] Björnmalm M, Thurecht KJ, Michael M, Scott AM, Caruso F (2017). Bridging bio-nano science and cancer nanomedicine. ACS Nano.

[26] Shi J, Kantoff PW, Wooster R, Farokhzad OC (2017). Cancer nanomedicine: progress, challenges and opportunities. Nat. Rev. Cancer.

[27] Sheeran PS, Dayton PA (2012). Phase-change contrast agents for imaging and therapy. Curr. Pharm. Des..

[28] Wang X, Chen H, Chen Y, Ma M, Zhang K, Li F, Zheng Y, Zeng D, Wang Q, Shi J (2012). Perfluorohexane-encapsulated mesoporous silica nanocapsules as enhancement agents for highly efficient high intensity focused ultrasound (HIFU). Adv. Mater..

[29] Liberman A, Wu Z, Barback CV, Viveros R, Blair SL, Ellies LG, Vera DR, Mattrey RF, Kummel AC, Trogler WC (2013). Color doppler ultrasound and gamma imaging of intratumorally injected 500 nm iron-silica nanoshells. ACS Nano.

[30] Zhang K, Chen H, Li F, Wang Q, Zheng S, Xu H, Ma M, Jia X, Chen Y, Mou J, Wang X, Shi J (2014). A continuous tri-phase transition effect for HIFU-mediated intravenous drug delivery. Biomaterials.

[31] Min KH, Min HS, Lee HJ, Park DJ, Yhee JY, Kim K, Kwon IC, Jeong SY, Silvestre OF, Chen X, Hwang YS, Kim EC, Lee SC (2015). pH-controlled gas-generating mineralized nanoparticles: a theranostic agent for ultrasound imaging and therapy of cancers. ACS Nano..

[32] Li Y, Chen Y, Du M, Chen Z-Y (2018). Ultrasound technology for molecular imaging: from contrast agents to multimodal imaging. ACS Biomater. Sci. Eng..

[33] Il Yoon Y, Tang W, Chen X (2017). Ultrasound-mediated diagnosis and therapy based on ultrasound contrast agents. Small Methods..

[34] Yildirim A, Chattaraj R, Blum NT, Goldscheitter GM, Goodwin AP (2016). Stable encapsulation of air in mesoporous silica nanoparticles: fluorocarbon-free nanoscale ultrasound contrast agents. Adv. Healthc. Mater..

[35] Tamarov K, Sviridov A, Xu W, Malo M, Andreev V, Timoshenko V, Lehto V-P (2017). Nano air seeds trapped in mesoporous janus nanoparticles facilitate cavitation and enhance ultrasound imaging. ACS Appl. Mater. Interfaces.

[36] Jin Q, Lin C-Y, Kang S-T, Chang Y-C, Zheng H, Yang C-M, Yeh C-K (2017). Superhydrophobic silica nanoparticles as ultrasound contrast agents. Ultrason. Sonochem..

[37] Kwan JJ, Myers R, Coviello CM, Graham SM, Shah AR, Stride E, Carlisle RC, Coussios CC (2015). Ultrasound-propelled nanocups for drug delivery. Small.

[38] Thomas RG, Jonnalagadda US, Kwan JJ (2019). Biomedical applications for gas-stabilizing solid cavitation agents. Langmuir.

[39] Montoya Mira J, Wu L, Sabuncu S, Sapre A, Civitci F, Ibsen S, Esener S, Yildirim A (2020). Gas-stabilizing sub-100 nm mesoporous silica nanoparticles for ultrasound theranostics. ACS Omega.

[40] Ho Y-J, Wu C-H, Jin Q, Lin C-Y, Chiang P-H, Wu N, Fan C-H, Yang C-M, Yeh C-K (2020). Superhydrophobic drug-loaded mesoporous silica nanoparticles capped with β-cyclodextrin for ultrasound image-guided combined antivascular and chemo-sonodynamic therapy. Biomaterials.

[41] Blum NT, Gyorkos CM, Narowetz SJ, Mueller EN, Goodwin AP (2019). Phospholipid-coated hydrophobic mesoporous silica nanoparticles enhance thrombectomy by high-intensity focused ultrasound with low production of embolism-inducing clot debris. ACS Appl. Mater. Interfaces.

[42] Mannaris C, Bau L, Grundy M, Gray M, Lea-Banks H, Seth A, Teo B, Carlisle R, Stride E, Coussios CC (2019). Microbubbles, nanodroplets and gas-stabilizing solid particles for ultrasound-mediated extravasation of unencapsulated drugs: an exposure parameter optimization study. Ultrasound Med. Biol..

[43] Grundy M, Bau L, Hill C, Paverd C, Mannaris C, Kwan J, Crake C, Coviello C, Coussios C, Carlisle R (2021). Improved therapeutic antibody delivery to xenograft tumors using cavitation nucleated by gas-entrapping nanoparticles. Nanomedicine.

[44] Jin Q, Lin C-Y, Chang Y-C, Yang C-M, Yeh C-K (2018). Roles of textural and surface properties of nanoparticles in ultrasound-responsive systems. Langmuir.

[45] Kwan JJ, Lajoinie G, de Jong N, Stride E, Versluis M, Coussios CC (2016). Ultrahigh-speed dynamics of micrometer-scale inertial cavitation from nanoparticles. Phys. Rev. Appl..

[46] Yildirim A, Shi D, Roy S, Blum NT, Chattaraj R, Cha JN, Goodwin AP (2018). Nanoparticle-mediated acoustic cavitation enables high intensity focused ultrasound ablation without tissue heating. ACS Appl. Mater. Interfaces.

[47] Mannaris C, Teo BM, Seth A, Bau L, Coussios C, Stride E (2018). Gas-stabilizing gold nanocones for acoustically mediated drug delivery. Adv. Healthc. Mater..

[48] Zhao Y, Zhu Y, Fu J, Wang L (2014). Effective cancer cell killing by hydrophobic nanovoid-enhanced cavitation under safe low-energy ultrasound. Chem. Asian J..

[49] Zhao Y, Zhu Y (2014). Synergistic cytotoxicity of low-energy ultrasound and innovative mesoporous silica-based sensitive nanoagents. J. Mater. Sci..

[50] Caupin F, Herbert E (2006). Cavitation in water: a review. Comptes Rendus Phys..

[51] Herbert E, Balibar S, Caupin F (2006). Cavitation pressure in water. Phys. Rev. E..

[52] Apfel RE (1984). Acoustic cavitation inception. Ultrasonics.

[53] Strasberg M (1959). Onset of ultrasonic cavitation in tap water. J. Acoust. Soc. Am..

[54] Apfel RE (1970). The role of impurities in cavitation-threshold determination. J. Acoust. Soc. Am..

[55] Crum LA (1979). Tensile strength of water. Nature.

[56] Harvey EN, Barnes DK, McElroy WD, Whiteley AH, Pease DC, Cooper KW (1944). Bubble formation in animals. I. Physical factors. J. Cell. Comp. Physiol..

[57] Atchley AA, Prosperetti A (1989). The crevice model of bubble nucleation. J. Acoust. Soc. Am..

[58] Arora M, Ohl C-D, Mørch KA (2004). Cavitation inception on microparticles: a self-propelled particle accelerator. Phys. Rev. Lett..

[59] Marschall HB, Mørch KA, Keller AP, Kjeldsen M (2003). Cavitation inception by almost spherical solid particles in water. Phys. Fluids.

[60] Borkent BM, Arora M, Ohl C-D (2007). Reproducible cavitation activity in water-particle suspensions. J. Acoust. Soc. Am..

[61] Gu Y, Li B, Chen M (2016). An experimental study on the cavitation of water with effects of SiO_2_ nanoparticles. Exp. Therm. Fluid Sci..

[62] Lohse D, Zhang X (2015). Surface nanobubbles and nanodroplets. Rev. Mod. Phys..

[63] Seddon JRT, Kooij ES, Poelsema B, Zandvliet HJW, Lohse D (2011). Surface bubble nucleation stability. Phys. Rev. Lett..

[64] German SR, Wu X, An H, Craig VSJ, Mega TL, Zhang X (2014). Interfacial nanobubbles are leaky: permeability of the gas/water interface. ACS Nano.

[65] Zhang XH, Maeda N, Craig VSJ (2006). Physical properties of nanobubbles on hydrophobic surfaces in water and aqueous solutions. Langmuir.

[66] Chan CU, Ohl C-D (2012). Total-internal-reflection-fluorescence microscopy for the study of nanobubble dynamics. Phys. Rev. Lett..

[67] Borkent BM, Gekle S, Prosperetti A, Lohse D (2009). Nucleation threshold and deactivation mechanisms of nanoscopic cavitation nuclei. Phys. Fluids.

[68] Belova V, Krasowska M, Wang D, Ralston J, Shchukin DG, Möhwald H (2013). Influence of adsorbed gas at liquid/solid interfaces on heterogeneous cavitation. Chem. Sci..

[69] Belova V, Gorin DA, Shchukin DG, Möhwald H (2010). Selective ultrasonic cavitation on patterned hydrophobic surfaces. Angew. Chemie Int. Ed..

[70] Jin Q, Kang S-T, Chang Y-C, Zheng H, Yeh C-K (2016). Inertial cavitation initiated by polytetrafluoroethylene nanoparticles under pulsed ultrasound stimulation. Ultrason. Sonochem..

[71] Peng H, Hampton MA, Nguyen AV (2013). Nanobubbles do not sit alone at the solid-liquid interface. Langmuir.

[72] Li D, Pan Y, Zhao X, Bhushan B (2016). Study on nanobubble-on-pancake objects forming at polystyrene/water interface. Langmuir.

[73] Wu Q, Zhang F, Pan X, Huang Z, Zeng Z, Wang H, Jiao J, Xiong X, Bai L, Zhou D, Liu H (2021). Surface wettability of nanoparticle modulated sonothrombolysis. Adv. Mater..

[74] Larina IV, Evers BM, Ashitkov TV, Bartels C, Larin KV, Esenaliev RO (2005). Enhancement of drug delivery in tumors by using interaction of nanoparticles with ultrasound radiation. Technol. Cancer Res. Treat..

[75] I.V. Larina, C. Bartels, K.V. Larin, R.O. Esenaliev, *Cavitation-Induced Drug Delivery in Tumors for Cancer Chemotherapy: Phantom Studies*, In D.D. Duncan, S.L. Jacques, P.C. Johnson (eds) Laser-Tissue Interaction XII: Photochemical, Photothermal, and Photomechanical, vol. 4257 (2001), SPIE, p. 385

[76] R.O. Esenaliev, I.V. Larina, Y. Ivanova, T.V. Ashitkov, R. Thomas, and B.M. Evers, *Cavitation-Induced Drug Delivery in Tumors for Cancer Chemotherapy: Animal Studies*, In Laser-Tissue Interaction XII: Photochemical, Photothermal, and Photomechanical, vol. 4257 (2001), p. 393

[77] S.J. Wagstaffe, H.A. Schiffter, M. Arora, and C.-C. Coussios, *Sonosensitive Nanoparticles for Controlled Instigation of Cavitation and Drug Delivery by Ultrasound*, In AIP Conference Proceedings, vol. 1481 (2012), American Institute of Physics, p. 426.

[78] Yildirim A, Chattaraj R, Blum NT, Goodwin AP (2016). Understanding acoustic cavitation initiation by porous nanoparticles: toward nanoscale agents for ultrasound imaging and therapy. Chem. Mater..

[79] Blum NT, Yildirim A, Gyorkos C, Shi D, Cai A, Chattaraj R, Goodwin AP (2019). Temperature-responsive hydrophobic silica nanoparticle ultrasound contrast agents directed by phospholipid phase behavior. ACS Appl. Mater. Interfaces.

[80] Yildirim A, Chattaraj R, Blum NT, Shi D, Kumar K, Goodwin AP (2017). Phospholipid capped mesoporous nanoparticles for targeted high intensity focused ultrasound ablation. Adv. Healthc. Mater..

[81] Su X, Thomas RG, Bharatula LD, Kwan JJ (2019). Remote targeted implantation of sound-sensitive biodegradable multi-cavity microparticles with focused ultrasound. Sci. Rep..

[82] Zhang L, Belova V, Wang H, Dong W, Möhwald H (2014). Controlled cavitation at nano/microparticle surfaces. Chem. Mater..

[83] Ancona A, Troia A, Garino N, Dumontel B, Cauda V, Canavese G (2020). Leveraging re-chargeable nanobubbles on amine-functionalized ZnO nanocrystals for sustained ultrasound cavitation towards echographic imaging. Ultrason Sonochem.

[84] Wang X, Yu X, Wang X, Qi M, Pan J, Wang Q (2019). One-step nanosurface self-assembly of D-peptides renders bubble-free ultrasound theranostics. Nano Lett..

[85] Wang X, Qiao L, Yu X, Wang X, Jiang L, Wang Q (2019). Controllable formation of ternary inorganic-supramolecular-polymeric hydrogels by amidation-fueled self-assembly and enzymatic post-cross-linking for ultrasound theranostic. ACS Biomater. Sci. Eng..

[86] Hiltl P, Grebner A, Fink M, Rupitsch S, Ermert H, Lee G (2019). Inertial cavitation of lyophilized and rehydrated nanoparticles of poly(L-Lactic Acid) at 835 KHz and 1.8 MPa ultrasound. Sci. Rep..

[87] Su X, Rakshit M, Das P, Gupta I, Das D, Pramanik M, Ng KW, Kwan J (2021). Ultrasonic implantation and imaging of sound-sensitive theranostic agents for the treatment of arterial inflammation. ACS Appl. Mater. Interfaces.

[88] Jonnalagadda US, Su X, Kwan JJ (2021). Nanostructured TiO2 cavitation agents for dual-modal sonophotocatalysis with pulsed ultrasound. Ultrason. Sonochem..

[89] Matsumura Y, Maeda H (1986). A new concept for macromolecular therapeutics in cancer chemotherapy: mechanism of tumoritropic accumulation of proteins and the antitumor agent smancs. Cancer Res..

[90] Iyer AK, Khaled G, Fang J, Maeda H (2006). Exploiting the enhanced permeability and retention effect for tumor targeting. Drug Discov. Today.

[91] Skrabalak SE, Chen J, Sun Y, Lu X, Au L, Cobley CM, Xia Y (2008). Gold nanocages: synthesis, properties, and applications. Acc. Chem. Res..

[92] Shen D, Yang J, Li X, Zhou L, Zhang R, Li W, Chen L, Wang R, Zhang F, Zhao D (2014). Biphase stratification approach to three-dimensional dendritic biodegradable mesoporous silica nanospheres. Nano Lett..

[93] Deckers R, Merckel LG, Denis De Senneville B, Schubert G, Köhler M, Knuttel FM, Mali WPTM, Moonen CTW, Van Den Bosch MAAJ, Bartels LW (2015). Performance analysis of a dedicated breast MR-HIFU system for tumor ablation in breast cancer patients. Phys. Med. Biol..

[94] Illing RO, Kennedy JE, Wu F, ter Haar GR, Protheroe AS, Friend PJ, Gleeson FV, Cranston DW, Phillips RR, Middleton MR (2005). The safety and feasibility of extracorporeal high-intensity focused ultrasound (HIFU) for the treatment of liver and kidney tumours in a Western population. Br. J. Cancer.

[95] Golan R, Bernstein A, Sedrakyan A, Daskivich TJ, Du DT, Ehdaie B, Fisher B, Gorin MA, Grunberger I, Hunt B, Jiang HH, Kim HL, Marinac-Dabic D, Marks LS, McClure TD, Montgomery JS, Parekh DJ, Punnen S, Scionti S, Viviano CJ, Wei JT, Wenske S, Wysock JS, Rewcastle J, Carol M, Oczachowski M, Hu JC (2018). Development of a nationally representative coordinated registry network for prostate ablation technologies. J. Urol..

[96] Devarakonda SB, Myers MR, Lanier M, Dumoulin C, Banerjee RK (2017). Assessment of gold nanoparticle-mediated-enhanced hyperthermia using MR-guided high-intensity focused ultrasound ablation procedure. Nano Lett..

[97] Hijnen N, Kneepkens E, de Smet M, Langereis S, Heijman E, Grüll H (2017). Thermal combination therapies for local drug delivery by magnetic resonance-guided high-intensity focused ultrasound. Proc. Natl. Acad. Sci..

[98] Tempany CMC, McDannold NJ, Hynynen K, Jolesz FA (2011). Focused ultrasound surgery in oncology: overview and principles. Radiology.

[99] Khokhlova TD, Monsky WL, Haider YA, Maxwell AD, Wang Y-N, Matula TJ (2016). Histotripsy liquefaction of large hematomas. Ultrasound Med. Biol..

[100] Khokhlova VA, Fowlkes JB, Roberts WW, Schade GR, Xu Z, Khokhlova TD, Hall TL, Maxwell AD, Wang Y-N, Cain CA (2015). Histotripsy methods in mechanical disintegration of tissue: towards clinical applications. Int. J. Hyperth..

[101] Hall TL, Hempel CR, Wojno K, Xu Z, Cain CA, Roberts WW (2009). Histotripsy of the prostate: dose effects in a chronic canine model. Urology.

[102] Liberman A, Wu Z, Barback CV, Viveros RD, Wang J, Ellies LG, Mattrey RF, Trogler WC, Kummel AC, Blair SL (2014). Hollow iron-silica nanoshells for enhanced high intensity focused ultrasound. J. Surg. Res..

[103] Bhatnagar S, Kwan JJ, Shah AR, Coussios C-C, Carlisle RC (2016). Exploitation of sub-micron cavitation nuclei to enhance ultrasound-mediated transdermal transport and penetration of vaccines. J. Control. Release.

[104] Myers R, Coviello C, Erbs P, Foloppe J, Rowe C, Kwan J, Crake C, Finn S, Jackson E, Balloul J-M, Story C, Coussios C, Carlisle R (2016). Polymeric cups for cavitation-mediated delivery of oncolytic vaccinia virus. Mol. Ther..

[105] Myers R, Grundy M, Rowe C, Coviello C, Bau L, Erbs P, Foloppe J, Balloul J-M, Story C, Coussios C, Carlisle R (2018). Ultrasound-mediated cavitation does not decrease the activity of small molecule, antibody or viral-based medicines. Int. J. Nanomed..

[106] Paris JL, Mannaris C, Cabañas MV, Carlisle R, Manzano M, Vallet-Regí M, Coussios CC (2018). Ultrasound-mediated cavitation-enhanced extravasation of mesoporous silica nanoparticles for controlled-release drug delivery. Chem. Eng. J..

[107] Lafond M, Yoshizawa S, Umemura S (2019). Sonodynamic therapy: advances and challenges in clinical translation. J. Ultrasound Med..

[108] Costley D, Mc Ewan C, Fowley C, McHale AP, Atchison J, Nomikou N, Callan JF (2015). Treating cancer with sonodynamic therapy: a review. Int. J. Hyperth..

[109] Gong Z, Dai Z (2021). Design and challenges of sonodynamic therapy system for cancer theranostics: from equipment to sensitizers. Adv. Sci..

[110] Miller DL, Smith NB, Bailey MR, Czarnota GJ, Hynynen K, Makin IRS (2012). Overview of therapeutic ultrasound applications and safety considerations. J. Ultrasound Med..

[111] Carstensen EL (1987). Acoustic cavitation and the safety of diagnostic ultrasound. Ultrasound Med. Biol..

[112] Galluzzi L, Buqué A, Kepp O, Zitvogel L, Kroemer G (2017). Immunogenic cell death in cancer and infectious disease. Nat. Rev. Immunol..

